# Genome Analyses Support Global Effective Panmixia in Sperm Whales

**DOI:** 10.3390/biology15181619

**Published:** 2026-09-14

**Authors:** Muhammad Basil Ali, Menno J. de Jong, Ulfur Arnason, Biagio Violi, Céline Tardy, Filipe Alves, Marina Arregui, Rui Prieto, Pablo Covelo, Daniel Engelhaupt, A. Rus Hoelzel, Axel Janke

**Affiliations:** 1 Senckenberg Biodiversity and Climate Research Centre (BiK-F), Georg-Voigt-Strasse 14–16, 60325 Frankfurt am Main, Germany; muhammad.ali@senckenberg.de (M.B.A.); menno.de-jong@senckenberg.de (M.J.d.J.); 2 Department of Neurosurgery, Skane University Hospital in Lund, 222 42 Lund, Sweden; 3 Menkab: il respiro del mare Association, Lungomare Matteotti 1, 17100 Savona, Italy; 4 WWF France, 6 rue des Fabres, 13001 Marseille, France; 5 MARE—Marine and Environmental Sciences Centre, ARNET—Aquatic Research Network, Agência Regional para o Desenvolvimento da Investigação, Tecnologia e Inovação (ARDITI), Edifício Madeira Tecnopolo, Piso 2, Caminho da Penteada, 9020-105 Funchal, Portugal; 6 Faculty of Life Sciences, University of Madeira, Campus Universitário da Penteada, 9020-105 Funchal, Portugal; 7 Veterinary Histology and Pathology, Atlantic Center for Cetacean Research (CAIC), Institute of Animal Health and Food Safety (IUSA), Veterinary School, University of Las Palmas de Gran Canaria (ULPGC), Trasmontaña s/n, 35413 Arucas, Spain; 8 Institute of Marine Sciences—Okeanos, Institute of Marine Research—IMAR, University of the Azores, Rua Professor Doutor Frederico Machado 4, 9901-862 Horta, Portugal; 9 Coordinadora para o Estudo dos Mamíferos Mariños (CEMMA), Camiño do Ceán 2, 36350 Nigrán, Spain; 10 HDR Inc., 701 Independence Circle, Virginia Beach, VA 23455, USA; 11 Department of Biosciences, Durham University, South Road, Durham DH1 3LE, UK; 12 Institute for Ecology, Evolution and Diversity, Goethe University, Max-von-Laue-Strasse 9, 60438 Frankfurt am Main, Germany

**Keywords:** panmixia, conservation genetics, population differentiation, cetacea

## Abstract

Unlike on land, the oceans present few biogeographic barriers to hinder migration and mate choice. One might therefore expect random mating across entire species ranges, yet this is rarely observed. Even the largest and most mobile marine species like whales often maintain basin-specific lineages. Here, we test whether sperm whales form a single effectively panmictic gene pool. We use the term effective panmixia to refer to the absence of detectable genome-wide allele-frequency differentiation, as opposed to equal mating probabilities among all individuals. We compiled data of 45 whole genomes (of which 41 newly sequenced) to perform phylogenetic and demographic analyses. The analyses uncover a remarkable lack of global population structure: sperm whales from the Atlantic, Pacific, and Indian Oceans form a single, genetically homogeneous population, suggesting global effective panmixia, with no significant differences in allele frequency. The only exception is the population of the Mediterranean Sea, which forms a distinct cluster, indicating past or ongoing geographic isolation and genetic drift owing to a reduced effective population size. The lack of general population structure elsewhere across the species range highlights the extraordinary global connectivity of this species. This global connectivity may buffer sperm whale populations from loss of variation through genetic drift, whereas the differentiated Mediterranean population remains a regional conservation priority.

## Simple Summary

Genomic analysis of 45 globally sampled sperm whales reveals an absence of global population structure except for a weak genetic discontinuity at the Strait of Gibraltar, making individuals in the North Atlantic more differentiated from conspecifics in the Mediterranean Sea than from conspecifics in the Indian and Pacific Ocean. These findings indicate global effective panmixia across the world’s oceans—a rare phenomenon in marine vertebrates.

## 1. Introduction

Genetic connectivity, drift, selection, isolation, and hybridization are central processes driving differentiation within species. Populations may diverge genetically through these mechanisms, frequently via spatial separation (allopatry), but also through ecological or behavioral isolation, ultimately leading to the formation of new species [1]. Marine environments are generally characterized by a lack of obvious physical barriers [2], and many marine taxa show strong population structure [3,4,5,6], and yet global panmixia, as a genome-wide absence of stable population differentiation across an entire species range, remains exceptionally rare [7].

Fisher defined panmixia as random mating among all potential partners [8]. Genetic studies usually assess departures from panmixia through allele-frequency differentiation [9]. Wright noted [10] that allele frequencies can remain homogeneous across large ranges despite limited direct encounters. We therefore distinguish true panmixia (equal mating probabilities) from effective panmixia (no detectable allele-frequency differences among groups).

The super-family, Physeteroidea (the sperm whale, pygmy sperm whale, *Kogia breviceps*, and dwarf sperm whale, *K. sima*) diverged from other Odontocete (toothed) whales about 25 million years ago (Mya) [11]. As a cosmopolitan species, the sperm whale (*Physeter macrocephalus*) occurs in all oceans, but unlike in many other whales [3,12], there is to date little evidence for strong population structuring. Indeed, there is evidence for close kinship found among sperm whales either side of the North Atlantic Ocean [13]. Only the population of the Mediterranean sperm whales is recognized to be genetically somewhat distinct from the North Atlantic (FST = 0.03–0.04), which is likely caused by restricted gene flow through the Strait of Gibraltar [14,15]. This raises the question of whether the sperm whale may be effectively panmictic and has a common gene pool across its range.

Sperm whales have strongly sex- and age-structured life histories. Females and calves live in stable matrilineal social units with site fidelity and culturally transmitted vocal clans, whereas mature males disperse widely and visit lower-latitude female groups for mating [16,17,18,19]. Female philopatry is not necessarily absolute across longer timescales; because mtDNA is maternally inherited, persistent female site fidelity is expected to be expressed most clearly in mitochondrial rather than biparentally inherited nuclear variation. Direct mate choice is difficult to observe at sea, and mating should not be interpreted as behaviorally random; male competition, female social context, and repeated associations may influence reproductive opportunities. This distinction supports our use of effective rather than strict panmixia.

The historical impact of commercial whaling, halted from the 1985 season onwards, decimated sperm whale populations globally to about 43% of the original population size [20]. Despite this dramatic decline, there are globally still close to 850,000 sperm whales that can be expected to have preserved a rich genetic diversity and their original population structure. Because sperm whales have diverse social behavior in their sympatric clans [16,17], populations may have differentiated and adapted to local environments.

Yet for sperm whales, studies find limited genetic differentiation and diversity across ocean basins using mtDNA, microsatellites, and a few single-nucleotide polymorphisms [19,21,22]. By comparison, within blue whales and orcas, genomic studies have found distinct populations that are the basis for defining sub-species for blue whales, notably the pygmy blue whale [23] and the Pacific blue whale [3]. In the killer whale (*Orcinus orca*), distinct populations [6], even in sympatry [12], can be defined genetically, matching behavioral differences, like food preferences [24]. Similarly, genomic work in fin whales also indicates population structuring despite high mobility [4].

Only the Mediterranean sperm whale population seems to be genetically distinct [15]. This population is estimated to contain fewer than 2500 individuals and is classified as Endangered by the IUCN [25]. However, genomic analyses of ddRADseq data from Atlantic and Mediterranean individuals did indicate some ongoing gene flow through the Strait of Gibraltar [15].

**Figure 1:**
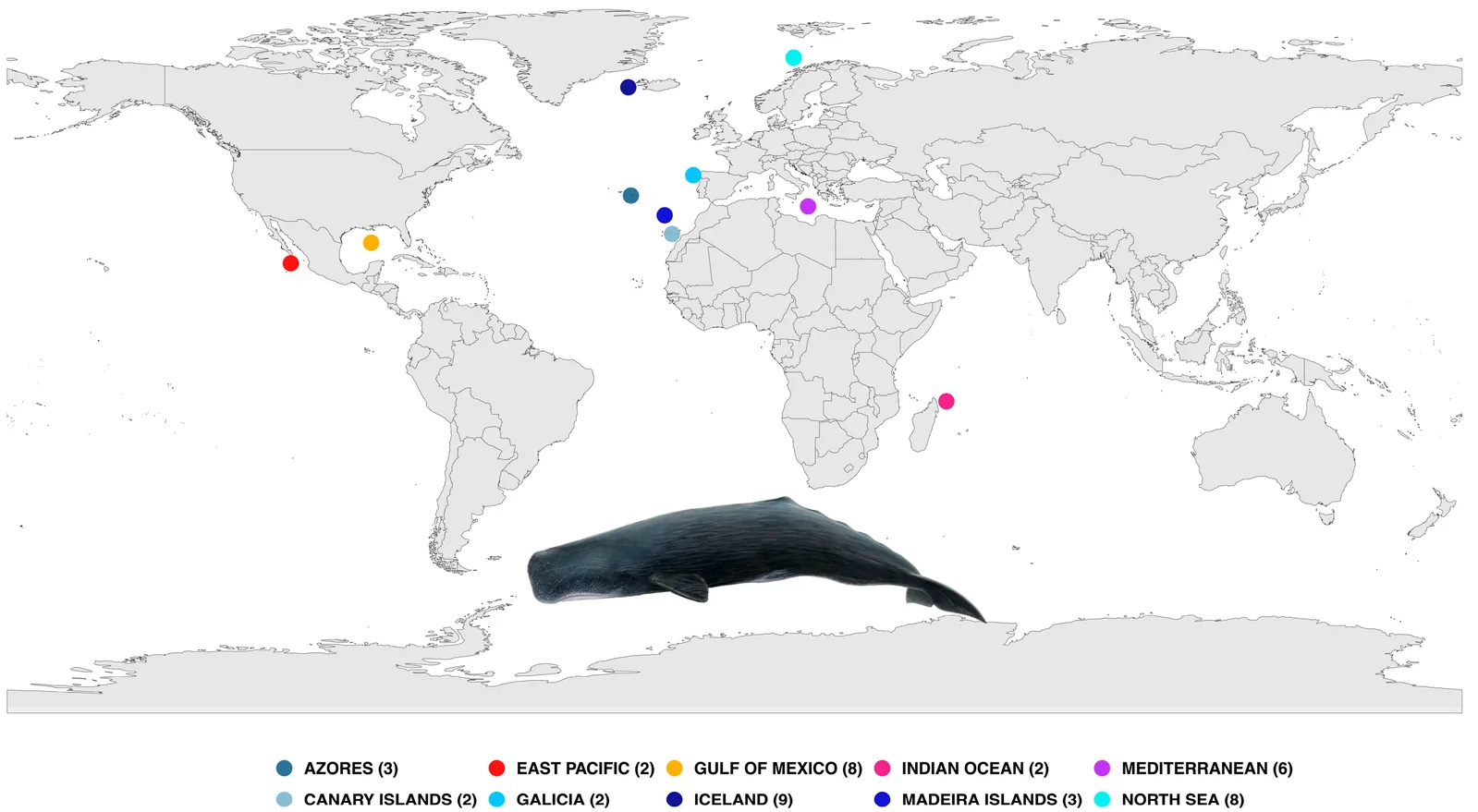
Geographic distribution of sampled sperm whale individuals across ten distinct marine regions. Sampled regions include the following: Azores (*n* = 3), Canary Islands (*n* = 2), East Pacific (*n* = 2), Galicia (*n* = 2), Gulf of Mexico (*n* = 8), Iceland (*n* = 9), Indian Ocean (*n* = 2), Madeira (*n* = 3), Mediterranean (*n* = 6), and North Sea (*n* = 8).

To date, genetic studies on sperm whale populations based on mitochondrial DNA (mtDNA) and microsatellite DNA [19,21,26,27,28] have not convincingly resolved sperm whale differentiation across the oceans. We hypothesized that, except for the Mediterranean Sea, sperm whales form one effectively panmictic global gene pool. To test this hypothesis, we sequenced and analyzed 45 genomes (41 newly generated and four publicly available) representing major ocean basins (Figure 1) for an in-depth analysis of their genomic diversity and global population structure.

## 2. Materials and Methods

### 2.1. Sample Collection

A total of 41 sperm whale samples were collected by dart-biopsies from living individuals or by tissue collection from stranded individuals (Table S1). Biopsy samples from live animals were obtained using a standard remote dart-biopsy protocol [29]. In brief, cylindrical stainless-steel biopsy tips (6 mm diameter, 40 mm length) fitted with a foam stopper to limit penetration and ensure flotation were deployed using a 125–150 lb crossbow. Tips were sterilized before each sampling attempt, no retrieval line was attached to avoid entanglement, and floating darts were retrieved from the water as quickly as possible. Samples were taken by experienced personnel from apparently healthy animals, typically just below the dorsal ridge or from the peduncle, under the relevant legal permits. Additionally, four genomes (Table S2; two from the East Pacific Ocean and two from the Indian Ocean) were retrieved from NCBI’s SRA repository (four public genomes; 45 genomes in total) [30]. Among the individuals studied, eight were females, while the remainder were males. All samples were assigned to the single species *Physeter macrocephalus* from collection metadata and concordant mapping to the *P. macrocephalus* reference genome and mtDNA analyses; no sample showed evidence of assignment to *Kogia* or another cryptic lineage.

### 2.2. Ethics Statement

Samples were obtained from stranded animals or by biopsy under the applicable collection and handling permits. These include Galicia permits 541/2010 and 001/2014; Madeira permit 08-02/IFCN/2018–2019 FAU-MAD; Azores permits 20/2009/DRA, 30/2015/DRA and 80/2017/DRA; permission from the Spanish Ministry of Environment for Canary Islands strandings; and MMPA/ESA permit #0909-1465 (issued 14 June 1999) for Gulf of Mexico samples. Icelandic samples predate 1975. No institutional ethics committee was in place for the Azores samples, and no ethics committee was required for the Gulf of Mexico samples under the applicable permit.

DNA extraction followed standard chloroform-phenol protocols using tissue samples. Each sample was sequenced at a minimum depth of 10× coverage using an Illumina NovaSeq 6000 platform (Illumina, Inc., San Diego, CA, USA) at Novogene facilities in Cambridge, UK, and Munich, Germany.

### 2.3. Read Mapping and Genotype Calling

Raw sequencing reads were evaluated using FastQC v0.12.1 and MultiQC v1.30 [31] to assess quality. Processed reads were aligned to a female sperm whale reference genome (GCF_002837175.3) [32] with BWA-MEM v0.7.17 using default settings [33]. Only high-confidence alignments were retained (mapping quality ≥ 20 and alignment score > 100). Samtools v1.20 [34] removed reads with low mapping quality (<20), low alignment scores (<100), or discordant mapping (option: -f 0x2). Picard MarkDuplicates v3.3.0 [35] eliminated duplicate reads, with “REMOVE_DUPLICATES” set to TRUE.

Genotype likelihoods and variant calls were determined using the bcftools v1.20 mpileup and call pipeline [36] developed and implemented in previous studies [37,38,39]. Excessive mismatches were adjusted with the -C50 parameter of the mpileup command. In bcftools call, ploidy was assigned based on genomic region (mtDNA, autosomal, and X-chromosome) and individual sex. When sex information was unavailable, X-chromosomal mapping rates were used to infer sex. Indels were normalized using “bcftools norm-check-ref w.”

To minimize bias, genotype calling was performed using the “group-samples” option in bcftools, ensuring each individual was analyzed independently. Although this method may slightly increase genotype error rates in panmictic populations [40], heterozygosity comparisons validated its accuracy for datasets with unbalanced sample distributions from distinct populations [37]. This approach avoids population assignment assumptions, making results reproducible across datasets.

The bcftools filter pipeline removed sites with a read depth below five, an experimentally determined threshold balancing data retention and heterozygosity accuracy [37]. Autosomal sites with a combined read depth of 225–700 across individuals were retained (Figures S1 and S2), with a 5% missing data threshold. X-chromosomal sites were filtered for a depth range of 80–475 (Figure S3A,B). The combined-depth thresholds were centered on the expected total coverage across 45 genomes and evaluated against the empirical distributions in Supplementary Materials Figures S1 and S2. Because filtering used depth and missingness rather than allele state, it is not expected to preferentially remove divergent alleles, although repetitive, copy-number-variable, or consistently low-mappability regions may be under-represented.

### 2.4. Structure Analyses

Estimates of pairwise genetic dissimilarity, *E*(*p*), [41] were computed for each sample pair across autosomes and X-chromosomes, considering both polymorphic and monomorphic sites. The following rules applied for diploid individuals: *E*(*p*) = 0 for AA/AA; *E*(*p*) = 0.5 for AA/AT or AT/AT; *E*(*p*) = 0.75 for AT/CT; and *E*(*p*) = 1 for AA/TT or AC/GT. For haplodiploid data: *E*(*p*) = 0 for A/AA; *E*(*p*) = 0.5 for A/AT; and *E*(*p*) = 1 for A/TT. For haploid sequences: *p* = 0 for A/A; and *p* = 1 for A/T.

The resulting matrices of uncorrected genetic distances between individuals were used as input for both non-hierarchical analyses (i.e., principal coordinate analyses, or PCoA) and hierarchical cluster analyses (i.e., biological neighbor-joining, bioNJ), by running the functions ‘pcoa’ and ‘bionj’ of the R package ape v5.3. Because of the generally low variation in genetic distances across pairs of individuals, we opted to depict these multi-locus distance-based phylogenies in the unrooted format, as this format accentuates basal splits [41].

A genome-wide, SNP-based bootstrapped neighbor-joining phylogram was constructed in R, based on the Rogers genetic distance [42] among all sperm whale individuals, using the functions ‘rogers.dist’ and ‘aboot’ of the R package poppr v2.9.8 [43]. We created a phylogeny (i.e., species tree) using the software TreeMix v1.13, with default settings and the EastPacific population as outgroup, based on allele frequencies derived from the autosomal SNP dataset [44]. Node support was evaluated based on 1000 bootstrap repetitions; only branches with bootstrap values (BV) greater than 50% were retained for interpretation.

Genetic differentiation among sampling regions was assessed using two different Fst-methods: Hudson Fst and Weir and Cockerham’s Fst. The former was estimated from the E(p)-distance matrix using the ‘runDIST’ function of the R package SambaR v1.10. The latter was estimated from SNP data using the ‘stamppFst’ function of the R package StAMPP v1.6.3 [45]. Significance was evaluated using 500 bootstrap repetitions.

### 2.5. Run of Homozygosity (ROH) Analyses

The total number of retained homozygous and heterozygous sites per sample were counted on a sliding-window basis, using non-overlapping windows with a fixed size of 20 kb. The counting was performed using the Darwindow pipeline (no formal version; script used on 10 January 2025), which depends on the software Tabix/HTSlib v1.20 [46] for the extraction of genomic regions, and which subsequently converts the count data into estimates of observed heterozygosity (*He*) and run-of-homozygosity content (F_ROH_). Based on visual examination of the sensitivity of ROH-analyses to various settings, ROHs were defined as continuous regions of at least 200 kb (i.e., ≥10 adjacent windows of 20 kb) with an average *He* value below 0.05%. Genome-wide observed heterozygosity (*He*) for each individual was calculated as the proportion of retained callable autosomal sites that were heterozygous.

### 2.6. Mitogenome Phylogeny

Mitochondrial genomes were extracted from the short read sequencing data using GetOrganelle v1.7.7, a de novo assembly pipeline designed for recovering organelle genomes from high-throughput sequencing data [47]. Raw reads were filtered for quality using fastp v0.20.1 [48], and mtDNA sequences were assembled with the following parameters “*get_organelle_from_reads.py -1 forward.fq -2 reverse.fq -R 10 -k 21,45,65,85,105 -F animal_mt -o animal_mt_out*”. Following extraction, mitochondrial gene annotation was performed using MitoFish v2028.08 [49]. The extracted sequences were aligned using MAFFT v7 [50], via the MAFFT online server, employing the L-INS-I algorithm for high accuracy multiple sequence alignment. A maximum likelihood phylogeny was inferred with IQ-TREE v2.4.0 [51,52] using default parameters, then optionally linearized using the “chronoMPL” function in the R package “ape v5.3” [53], which applies the mean path length method [54]. Divergence times were estimated assuming a mtDNA mutation rate of 1.0–2.0 × 10^−8^ per site per year [55].

### 2.7. Admixture Analyses

For admixture assessments, we used a thinned SNP dataset in which autosomal SNPs were retained at 50 kb intervals using the --thin option of VCFtools v0.1.16 to reduce linkage disequilibrium (LD) among closely spaced markers.

PLINK v1.90 [56] was used to convert VCF data into PED/RAW and MAP/BIM formats, applying the options make-bed, recode A, chr-set 95, and allow-extra-chr. Further SNP management was conducted in R v4.5.0 using the SambaR package [57]. SNP data were imported into R and stored in a genlight object via the “read.PLINK” function in adegenet-2.1.1 [58], and subsequently filtered using SambaR’s “filterdata” function, with parameters: indmiss = 0.25, snpmiss = 0.2, min_mac = 2, and dohefilter = TRUE.

Ancestry coefficients were estimated using the LEA-2.8.0 package in R, implementing the “snmf” and “Q” functions with alpha = 10, tolerance = 0.00001, and 200 iterations [59]. The optimal number of clusters (K) was determined using the elbow method [59], evaluating cross-entropy scores for K values ranging from 2 to 12, each tested in 50 independent runs. The best K value was identified as the point where cross-entropy scores plateaued.

### 2.8. Demographic History (PSMC)

Historical effective population size (*Ne*) trajectories were inferred using the Pairwise Sequentially Markovian Coalescent (PSMC) model as implemented in PSMC v0.6.5-r67 [60]. Because PSMC is estimated from one diploid genome at a time, five geographically dispersed genomes with suitable coverage (9–23-fold) were randomly selected to compare trajectory concordance; the comparison was not treated as replicated population-level estimation. For each individual, consensus sequences were generated using bcftools v1.20 and converted into fastq format using vcfutils.pl vcf2fq, with minimum and maximum threshold of 5 and 20, respectively. Consensus sequences were then converted into PSMC input format using pq2psmcfa utility, with a base quality threshold of 20. PSMC was executed with the parameter settings of -N25 -t15 -r5 -p “2 + 2 + 25 × 2 + 4 + 6”. The latter setting was selected following recommendations in Hilgers et al. (2025) [61], who demonstrated that improper time intervals schemes may produce false peaks in inferred demographic history. Estimates of Ne over time were scaled assuming a mutation rate of 2.5 × 10^−8^ mutations per site per generation and generation time of 30 years [30].

### 2.9. Stairway Plot

To infer historical demographic trends, we employed Stairway Plot v2 [62], a coalescent-based method that models change in effective population size (Ne) over time using the site frequency spectrum (SFS), unlike PSMC, which uses patterns of heterozygosity along a single diploid genome, and is therefore expected to differ somewhat in recent-time resolution while providing a complementary estimate of the same underlying demographic history. The SFS was generated using the program easySFS (https://github.com/isaacovercast/easySFS; accessed on 2 December 2024) [63]. A folded SFS was used, as ancestral states could not be confidentially determined, following best practices for non-model organisms [62,63]. We performed this analysis on a filtered dataset containing 35 individuals only from the North Atlantic population. Specifically, we removed all individuals from the Mediterranean and from the Indian and East Pacific oceans to minimize confounding demographic signals due to geographic isolation. This ensured the analysis focused solely on the panmictic group of North Atlantic individuals. The data were downprojected to 56 (i.e., 28 diploid individuals) to account for variability in missing data across individuals [64].

The output SFS vectors from easySFS were used as input for Stairway Plot. We specified the use of a folded SFS and set the mutation rate at 2.5 × 10^−8^ per generation [15,30,65] with a generation time of 30 years [15,19,30,66]. The total number of sites (L) used in the blueprint was derived from the number of callable sites (~463 MB). We used a training data proportion of 0.67 and generated 200 input files per replicate to ensure robust bootstrap estimation and confidence interval construction.

## 3. Results

We generated whole-genome sequences (WGS) from 41 individuals, with sampling densest in the North Atlantic and Mediterranean Sea, achieving an average sequence coverage of ~10-fold (Supplementary Materials Tables S3 and S4). To enhance geographic representation, we incorporated four publicly available WGS datasets from the Indian and Pacific oceans, giving 45 genomes in total (Figure 1) [32].

**Figure 2:**
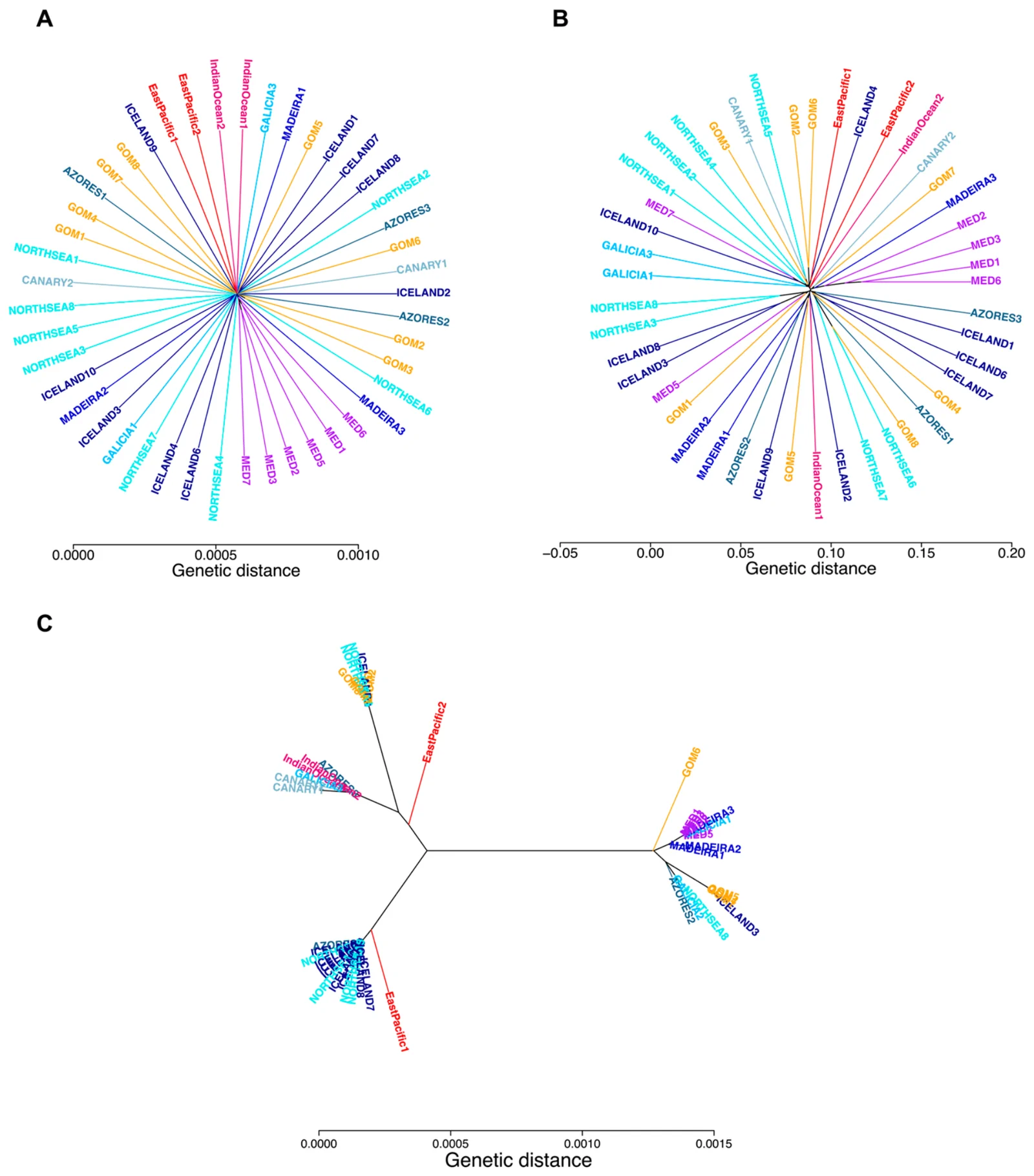
Genetic structure of sperm whale populations based on genome-wide nuclear and mitochondrial data. (**A**) Unrooted BioNJ tree based on genetic distances derived from genome-wide polymorphic autosomal SNPs. Branch lengths represent pairwise genetic dissimilarity, E(p), i.e., the proportion of genetic differences between individuals. The lack of prominent internal branches suggests effective panmixia (i.e., lack of allele frequency differences), with minimal divergence among populations. Only The Mediterranean population forms a weakly differentiated cluster. (**B**) Unrooted BioNJ tree based on genetic distances derived from X-chromosomal sites, with branch lengths expressed in the same genetic-distance units. The topology also shows panmixia, with minimal divergence among most populations. (**C**) Unrooted phylogenetic tree based on mitochondrial genome sequences, constructed using IQ-TREE. (Note: Colors correspond to Figure 1).

To assess population structure, we calculated genome-wide distances between all pairs of individuals as sequence dissimilarity, E(p), across 1.5 Gb of retained genomic sites, of which approximately 11 Mb were SNPs. Remarkably, we found that all individuals are roughly equidistant to each other, with distances ranging between 0.109–0.115% (Figure S4). This equidistance among samples remains regardless of whether including or excluding individuals from the Indian Ocean and Pacific Ocean, suggesting the absence of population structure, with individuals within ocean basins differing as much from each other as individuals from different basins. As a result, when constructing an unrooted bioNJ tree from the distance matrix, the resulting dendrogram is almost perfectly circular, with inner branches converging to zero (Figure 2A) [38,41]. The lack of meaningful and systematic difference in pairwise distances (Figure S5) is consistent with the virtually unstructured dendrogram. In line with this, no evidence for isolation by distance is apparent, as genomic dissimilarity does not increase with the large geographic separations represented by the Indian and Pacific samples.

**Figure 3:**
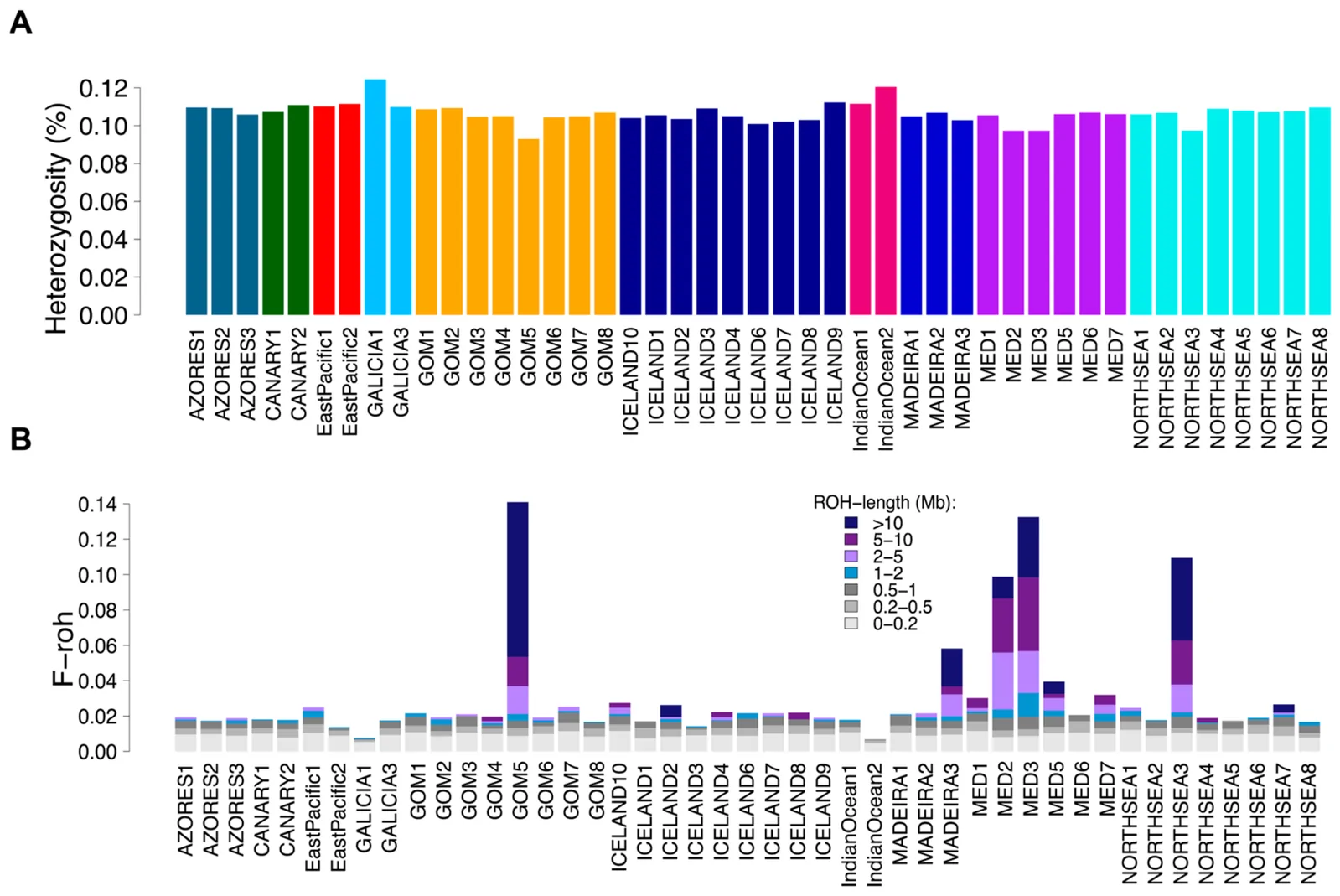
Genome-wide heterozygosity and regions of homozygosity in sperm whale populations. (**A**) Barplot displaying genome-wide observed heterozygosity for individual sperm whales across ten oceanic regions: Azores, Canary Islands, East Pacific, Galicia, Gulf of Mexico, Iceland, Indian Ocean, Madeira, Mediterranean, and North Sea. Each bar represents a single individual, with heterozygosity values expressed as the proportion of heterozygous sites per individual. The observed heterozygosity in sperm whales is approximately 0.08–0.10% in Mediterranean and ~0.11–0.12% in North Atlantic populations, which is comparable to He reported for blue whales (0.11%) [3], but higher than what is observed in humpback whales (0.03–0.05%) [68] and in killer whales (ranging from 0.029% to 0.058%) [24,69,70]. (**B**) Barplot illustrating individual runs of homozygosity (ROH) regions. ROHs are categorized by length, and stacked bars show the contribution of each ROH length class per individual. Outside the Mediterranean—only two individuals have a noticeable amount of long ROHs—inbreeding is limited. (Note: Colors correspond to Figure 1).

The only deviation from a global effective panmixia is a genetic discontinuity (Fst < 0.033) at the Strait of Gibraltar, corresponding to a subtle cluster of Mediterranean individuals. Genetic diversity and distances within these are slightly below the global average (Figure 3A; Figure S5), a pattern evident in the dendrogram (Figure 2A) as shortened terminal branches, which is consistent with reduced diversity from a bottleneck [41]. These findings suggest limited genetic exchange between the Mediterranean and other oceanic populations.

The global distance dendrogram (Figure 2A) matches the Neighbor-Net topology (Figure S6), showing virtually no internal branching, except among Mediterranean samples. This supports global effective panmixia, with only exceedingly weak regional clustering, such as in the Indian and East Pacific oceans. Including Kogia breviceps as an outgroup did not resolve additional geographic structure in either the circular or rectangular BioNJ representations (Figures S7 and S8).

Mediterranean sperm whales form a subtle genetic group, likely due to geographic isolation imposed by the narrow and shallow Strait of Gibraltar. Within this group, further sub-structuring appears to result from relatedness. For instance, the kinship analysis presented in Table S5 indicates that individuals MED1 and MED5 are likely related. We constructed the phylogram using the Rogers genetic distance with 1000 bootstrap replicates (Figure S9). The topology shows random placement of individuals with no clustering by sampling region. Even the branch that groups all Mediterranean individuals was not supported (BV < 50%). The Treemix phylogeny, inferred from autosomal allele frequencies, indicated very short internal branches (as measured in drift units), consistent with the absence of population structure (Figure S10).

The X-chromosome dendrogram (Figure 2B) is similar to the autosomal dendrogram, lacking pronounced internal branches, with an apparently random placing of individuals from vastly different oceanic basins. This further supports the absence of population subdivision that is old and consistent enough for lineage sorting. In this analysis, MED5 and MED7 are placed outside the main Mediterranean grouping among North Atlantic individuals, a pattern that may reflect gene flow or represent remnants of historical colonization events into the Mediterranean Sea. MED5 and MED7 passed the X-chromosomal depth and missing-data filters, so this placement is unlikely to be caused solely by low coverage. However, the very short internal branches and lack of node support mean that admixture cannot be distinguished from stochastic placement or incomplete lineage sorting.

A maximum likelihood phylogeny based on mtDNA (Figure 2C), also reveals no consistent geographic population structure, despite the relatively rapid fixation rate of mitochondrial lineages, which should capture population differentiation. As with nuclear data, the Mediterranean population stands out with a somewhat consistent phylogenetic grouping, some of them clustering with individuals from the Atlantic, suggesting some gene flow or incomplete lineage sorting. The individuals from the Gulf of Mexico (GOM) and the Indian Ocean group with individuals of the Canary Islands, suggesting long dispersal events linking these distant groups. The corresponding rooted mtDNA phylogram likewise shows limited geographic clustering (Figure S11).

Genome-wide heterozygosity levels across all individuals reach 0.12% (Figure 3A). Values overlap broadly among most sampled regions, whereas Mediterranean individuals occupy the lower end of the observed range (0.08–0.10%; Figure 3A). This pattern is consistent with stronger genetic drift and potential inbreeding in the Mediterranean population [15]. Lower heterozygosity can indicate loss of standing variation through drift or inbreeding and may reduce the capacity of a small population to respond to environmental change [67]. Because several regions are represented by only two or three individuals, these values are interpreted as individual observations rather than precise range-wide estimates.

Atlantic populations exhibit higher and relatively uniform heterozygosity (~0.11–0.12%), clustering near the upper end of the observed range, consistent with gene flow and the extensive migratory behavior of sperm whales. Exceptions include GOM5 and NORTHSEA3, which show lower heterozygosity values, similar to Mediterranean individuals. These outliers may indicate local demographic effects in this region leading to inbreeding and are not typical.

The distribution of runs of homozygosity (ROH) across individuals (Figure 3B) provides additional resolution into sperm whale genetic diversity and demographic history. ROHs were categorized by length, enabling inference of historical versus recent inbreeding events. Individuals from the Mediterranean, particularly MED2 and MED3, exhibit a marked enrichment of long ROH segments, consistent with their lower genome-wide heterozygosity and indicative of inbreeding and reduced effective population size. Chromosome-level heterozygosity profiles used to validate ROH calls are shown in Figure S12A,B.

**Figure 4:**
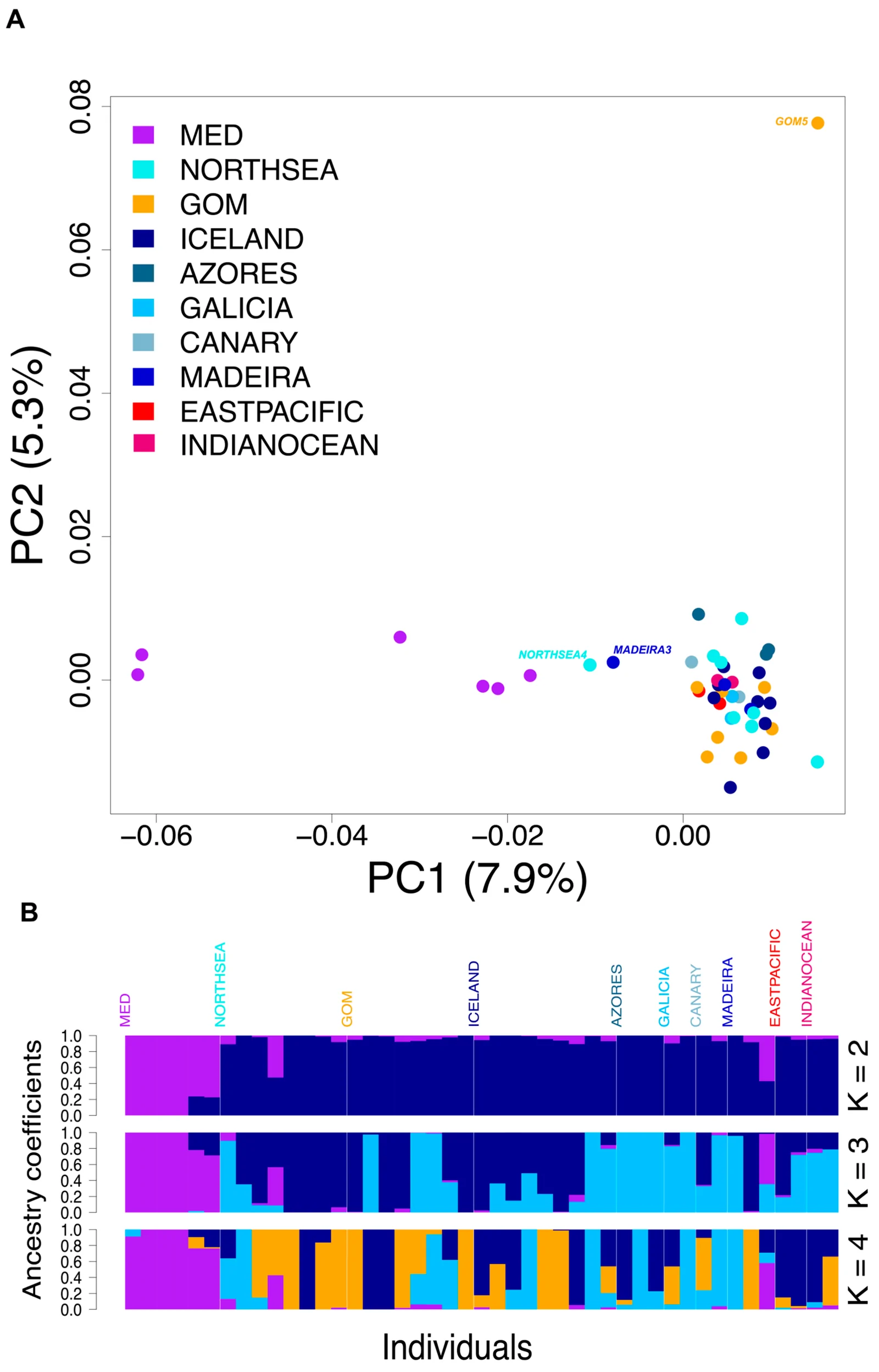
Principal coordinate analysis and admixture inference of sperm whale populations. (**A**) Principal coordinate analysis (PCoA) of autosomal SNP data from sperm whale individuals across all geographic regions.. Note that the primary axis separates Mediterranean samples, whereas Atlantic and Indian/Pacific samples cluster together. The positions of GOM5, MADEIRA3, and NORTHSEA4 are labeled to identify the individuals specifically discussed in the text. (**B**) Genetic structure inferred using sparse non-negative matrix factorization (sNMF) as implemented in the LEA package. Admixture proportions are shown for K = 2 to K = 4. While cross-entropy analysis identified K = 2 as the optimal number of ancestral populations, results for K > 2 are included to show the lack of additional structure at higher K-values. Each bar represents one individual, and the proportions of the different colors represent estimated ancestry coefficients. White vertical strips separate individuals belonging to different sampling populations and are included only as visual population boundaries.

Notably, NORTHSEA3 and GOM5 also display elevated ROH values relative to other individuals within their respective populations, paralleling their reduced heterozygosity levels. For GOM5, this pattern supports its outlier status in the principal coordinate analysis (PCoA; Figure 4A), suggesting occasional inbreeding or demographic isolation as in the Mediterranean individuals. Sequencing and mapping artifacts could not be identified.

The PCoA (Figure 4A) reveals substantial genetic overlap among individuals from geographically distant regions, including the Azores, Canary Islands, Gulf of Mexico, Iceland, and the North Sea. This widespread clustering underscores the low levels of population structuring and suggests genetic connectivity across global sperm whale populations, consistent with previous results. The same overall pattern remains after excluding the inbred individuals GOM5, NORTHSEA3, and NORTHSEA6 (Figure S13).

As expected, individuals from the Mediterranean form a separate cluster, consistent with reduced heterozygosity and elevated levels of long ROH segments. Within the Mediterranean cluster, sub-structuring consistent with previous dendrogram and kinship analyses (Figure 2A; Table S5) is evident.

An additional feature of interest is the positioning of individuals MADEIRA3 and NORTHSEA4, which cluster together in an intermediate space between the Mediterranean and broader Atlantic populations. Their proximity suggests potential shared ancestry or gene flow.

The LEA structure analysis (Figure 4B) is consistent with the PCoA results, revealing a global panmictic pattern of ancestry K = 2 to K = 4, except for population-level structuring for the Mediterranean population. Ancestry coefficients represent the estimated proportion of each individual assigned to each inferred ancestry component; values close to 1 indicate predominant assignment to one component, whereas intermediate values indicate mixed assignment. Interestingly, individuals MADEIRA3 and NORTHSEA4 exhibit partial assignment to the Mediterranean cluster, consistent with their intermediate positioning in PCoA plot and shared ancestry from North Atlantic individuals. Despite their slightly elevated Fst values, absolute genetic distances (Dxy) in Mediterranean individuals are marginally lower (Figure S5).

A Hudson Fst analysis on the genetic distinctiveness of Mediterranean individuals (Figure S14), yielded values below 0.02 consistent with a common genepool. Among populations, the lowest Fst value (0.005) is observed between the East Pacific and the North Sea, indicating strong genetic similarity despite maximal geographic separation. Similarly low values are observed for other geographically distant individuals. By contrast, the Hudson Fst values involving Mediterranean individuals are elevated (0.024 to 0.033) (Figure S14). This pattern is corroborated by Weir and Cockerham’s Fst estimates (Figures S15 and S16), which also show consistently higher levels of genetic differentiation between Mediterranean and North Atlantic populations.

**Figure 5:**
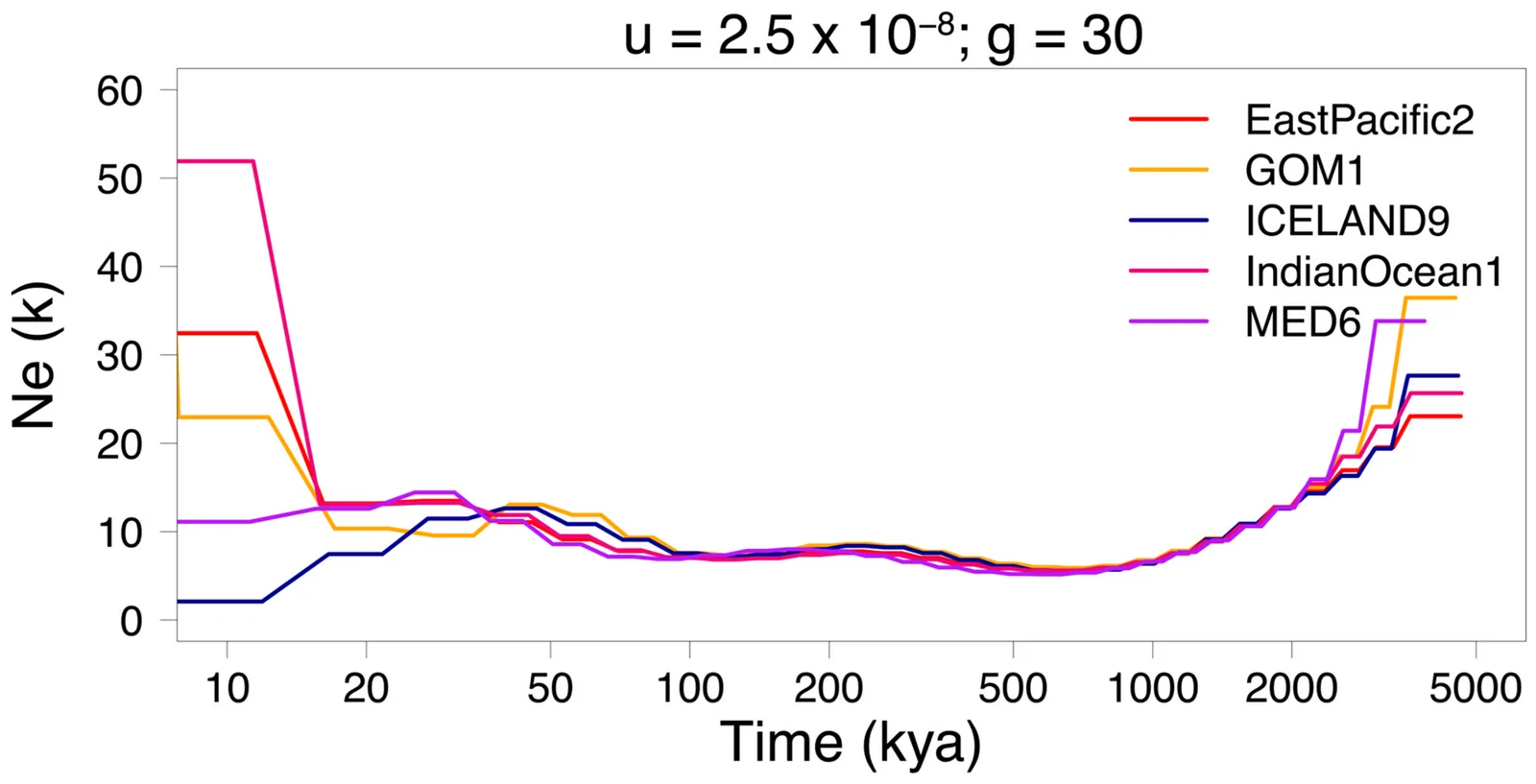
Demographic history of sperm whale individuals inferred using PSMC. PSMC-inferred trajectories of historical effective population size (Ne) for five individual sperm whales sampled from distinct oceanic regions. The analysis was performed assuming a mutation rate of 2.5 × 10^−8^ mutations per site per generation and a generation time of 30 years. The x-axis shows time (kyalog scale), and the y-axis shows Ne × 1000 on a linear scale. All individuals exhibit virtually identical demographic histories, characterized by a gradual decline in Ne beginning around 3 Mya and reaching a minimum between 100,000 and 200,000 ya, followed by a modest recent increase. The uniformity of trajectories across geographically dispersed individuals supports a near lack of population structure and is consistent with a panmictic population model. (Note: Colors correspond to Figure 1). Abbreviations: PSMC, Pairwise Sequentially Markovian Coalescent; Ne, effective population size; kya, thousand years ago.

The Pairwise Sequentially Markovian Coalescent (PSMC) analysis (Figure 5) on five sperm whale individuals (coverage 9–23-fold) from geographically distant populations is consistent with previous studies [30]. PSMC analyzes one diploid genome at a time; five geographically dispersed genomes with suitable coverage (9–23-fold) were therefore selected to compare trajectory concordance, not to provide replicated population-level estimates. All individuals, regardless of coverage, exhibit highly similar trajectories of their effective population size (Ne) over time, with a decline beginning around three million years ago (Mya) and then remaining constant throughout in the respective geographic areas with a negligible divergence between trajectories or specific demographic shifts. Moreover, a prominent bottleneck and subsequent population expansion is not evident. This pattern is also supported by the stairway plot demographic analysis (Figure S17).

## 4. Discussion

The complete absence of global population structure in sperm whales is remarkable. While earlier studies did not find pronounced population structure [19,21,26,27], they generally focused on a regional geographic range and used marker systems with lower resolution. A mitogenome study suggested that haplotypes were largely ocean-basin-specific [28] but could not be confirmed by whole mitogenome analyses in our increased dataset. Also, the analyses of the entire genome do not find differentiation, as evident from the equidistances and the population-genomic analyses, which indicates the absence of a Wahlund effect [21]. This lack of population differentiation across different ocean basins (Wahlund effect) is striking and exceedingly rare among marine vertebrates. It is consistent with the evidence found for admixture among sperm whales between the Gulf of Mexico and the North Sea [13].

This makes sperm whales a unique species relative to the rich population structuring in other large marine animals, such as humpback whales (*Megaptera novaeangliae*) which exhibit strong regional differentiation [68]. Similarly, blue whales (*Balaenoptera musculus*) demonstrate genetic structuring across ocean basins, likely due to their fidelity to specific feeding and breeding grounds [71] and are even differentiated into three subspecies with distinct genomic signatures [3]. Toothed whale species such as the cosmopolitan killer whales (*Orcinus orca*) show strong genetic differentiation too, often correlating with their ecological niches [24,69,70].

The observed lack of meaningful population structure could—theoretically—result from a recent range expansion, where all oceanic populations were repopulated from a single, localized refugial-stock, as indeed suggested by the shallow coalescence times of mtDNA haplotypes [28]. If such an extreme demographic event had occurred, classical population genetic theory implies that it must have taken place very recently to prevent the emergence of detectable genomic differentiation beyond the background noise introduced by sequencing errors and other artifacts. For illustration, assuming separation 10 kya ago (400 generations), Ne = 10,000, and μ = 2.5 × 10^−8^ per site per generation gives the following:

π = 4Neμ = 0.001

DXY = π + 2μt = 0.00102;  FST = (DXY − π)/DXY ≈ 0.02

This expected FST exceeds most empirical values observed for sperm whales (Hudson FST = 0.005–0.02; Figure S14).

Another difficulty of the range expansion hypothesis, as suggested by Lyrholm and Gyllensten, 1998 [72], is that the genomic data present no evidence for a recent population bottleneck and a subsequent increase [73], although we note that demographic inference close to the present may have limited resolution in a long-lived species with large effective population size and long generation time such as the sperm whale. Importantly, a recent population bottleneck is not supported by either PSMC or Stairway Plot analyses, which use different summaries of the genomic data and are therefore expected to differ in detail, especially near the present, while still being informative about the same broader demographic signal.

The uniformity of demographic trajectories in sperm whales is particularly striking when compared to PSMC results from other vertebrates, such as dolphins, flycatcher birds, or giraffe, where deviating Ne trajectories suggest independent evolution and lack of gene flow [5,74,75], and is therefore more consistent with shared demographic history and continued connectivity than with long-term demographic independence among ocean basins.

Thus, homogenizing gene flow emerges as the most plausible explanation consistent with the data. Although the strict theoretical definition of panmixia—all individuals have a strictly equal probability of mating—is unlikely to be met in natural populations, we propose a model of effective panmixia. In this framework, despite a natural gradient of mating probabilities that inversely correlates with geographic distance in sperm whales, gene flow remains sufficiently high to maintain a genetically homogeneous gene pool across the globe. It is possible that future, specialized methods may detect minor differentiation signals not recovered by the present analyses; however, if such a signal should exist, it would be too weak to challenge effective panmixia at the genome-wide level. Thus, female social philopatry reduces the number of maternal lineages locally, while occasional long-range dispersal limits lineage sorting. Male long-range dispersal could reduce or prevent nuclear differentiation.

Harvesting and beaching records, as well as tracking data, indicate that male and female sperm whales can be found as far south as the Antarctic [76], allowing them to migrate freely among the Atlantic and Indian and Pacific oceans [77,78,79].

Recent studies also report trans-oceanic kinship and high-latitude occurrence and migration in sperm whales [13,77,78,79], consistent with broad connectivity while not demonstrating random behavioral mating.

Given that females exhibit site fidelity, philopatry, and form stable social groups, while males undertake extensive long-distance movements [18,19], males are prime candidates for facilitating ocean-wide gene flow between the populations. However, the wide habitat ranges leave ample opportunity also for females and calves to migrate around the southern tip of South Africa, at 35° S. This implies that the populations of the Atlantic Ocean and the Indian Ocean can communicate through dispersal of either sex.

While satellite tracking data on movement patterns in the Indian Ocean and the southern Atlantic are lacking, it is known that female sperm whales and their offspring can be found year round off the coast of South Africa [80,81], connecting the Atlantic with the Indian ocean. This is unlike for the sailfish (*Istiophorus platypterus*) and the tiger shark (*Galeocerdo cuvier*), which are exothermic species and are generally not found far enough south and maintain distinct Atlantic and Indian/Pacific lineages [82,83].

Given the fast mutation and fixation rates [55], the mitochondrial genome remains a valuable and widely used genetic marker for population dynamics, even in the genomics era [84]. Some mtDNA studies reported maternal structuring in sperm whales, particularly in the Mediterranean and in the Gulf of Mexico, suggesting maternal philopatry [28]. The analysis of 175 individuals reported ocean-associated mitogenomic haplotypes, although diagnostic differences were limited [28]. These reported mtDNA patterns are compatible with philopatric female social units maintaining local mitochondrial and cultural structure, while long-distance male dispersal homogenizes nuclear variation. Consequently, dialect and mtDNA differences do not necessarily imply genome-wide reproductive isolation. The recently published Gulf of Mexico–western North Atlantic analysis [21,27,85] also emphasized declining relatedness with geographic distance, social-group structure, oil-spill exposure, and anthropogenic underwater noise as regional conservation concerns.

By contrast, mtDNA analyses in the current study do not support ocean-basin-specific clades. This conclusion remains unchanged when all publicly available 175 mitogenomes are integrated with our mitochondrial dataset. The enlarged mtDNA analysis still does not recover or hint at clean ocean-basin-specific clades (Figures S18 and S19). Furthermore, analyzing X-chromosome data, which are not affected by single-locus stochasticity, also reveals no population structure. This appears particularly insightful because the lower Ne and faster fixation rate of sex chromosomes [86,87] makes them a valuable tool for studying recent population differentiation [37,88].

Taken together, the combined mitochondrial and X-chromosomal results suggest that female philopatry in sperm whales is scale-dependent rather than absent. In our combined mitochondrial analysis, the 45 mitogenomes recovered from the present whole-genome dataset together with 175 published mitogenomes do not form clean ocean-basin-specific clades (Figures S18 and S19), while earlier regional studies detected female fidelity to coastal basins and local maternal structure [63,64]. We therefore interpret female philopatry as strongest within social units and recurrent regional habitats, but not sufficiently restrictive to produce deep matrilineal isolation among ocean basins. This interpretation is consistent with the lack of X-chromosomal structure and with Australian sperm whales from different historical whaling stocks being assigned to the same genetic population [17].

While the genomic data generally suggest absence of population structure, indicative of global effective panmixia, there is one intriguing exception: a genetic discontinuity at the Strait of Gibraltar leading to the Mediterranean clade. The lower levels of genetic diversity in the Mediterranean population, and the absence of elevated absolute distances (Dxy), indicate that these elevated Fst-value are caused by a loss of genetic diversity in the Mediterranean population, which in turn results from increased genetic drift associated with a population size reduction [15]. Just outside the Strait of Gibraltar, some individuals in the Atlantic are, based on relative genetic distance (Fst), even closer to individuals in the Indian and Pacific Ocean than to individuals in Mediterranean Sea.

A difference in genetic diversity between two neighboring populations can only exist if two populations are not communicating genetically. Population-genetic theory suggests that a single migrant per generation per effective population size (Nm ≥ 1) is in theory, and given enough time, sufficient to counteract the diversifying effects of genetic drift [89]. This suggests that the relatively narrow and shallow Strait of Gibraltar, or alternative factors such as resource preferences, are or have been acting as a barrier to gene flow between the Atlantic and Mediterranean Sea. Evidence for limited migration and genetic exchange from the structure and PCoA analyses suggests that this barrier may not be absolute, though.

The reduced population size of Mediterranean sperm whales is likely caused by the habitat geography, which compared to the main oceans provides relatively few deep sea areas for feeding [90]. The consequent presence of weak maternal structuring, somewhat reduced heterozygosity (0.10%), and increased ROH in the Mediterranean sperm whale suggest that this population may require closer genetic monitoring and targeted conservation strategies [15].

Previous work indicates restricted exchange through the Strait of Gibraltar and persistent regional structure within the Mediterranean [14,15]. A recent-colonization or founder scenario has been proposed from shallow mitochondrial coalescence, but the present genomic data do not estimate the timing or number of colonists [28,72]. The Mediterranean subpopulation is listed as Endangered [25], and Mediterranean physiography restricts the availability of suitable deep-water habitat [90]. Taken together, reduced heterozygosity and increased long ROH indicate greater sensitivity to drift and inbreeding. Regional management should prioritize genetic monitoring and reduction of ship strikes, entanglement, acoustic disturbance, and pollution [25]. Comparable concerns regarding oil exposure and anthropogenic underwater noise have been reported for the Gulf of Mexico–western North Atlantic [21,27,85]. Although a formal taxonomic recognition has been proposed, the weak nuclear differentiation and low absolute divergence observed here do not provide genomic support for a distinct subspecies [15].

In our view, the Mediterranean population represents the clearest case of ongoing regional differentiation in sperm whales. Restricted exchange, lower heterozygosity, increased long ROH and elevated relative FST show that genetic drift is stronger in this population, and fishery interactions and other anthropogenic mortality may further reduce its effective population size [22]. The reported smaller size of reproductive females may therefore be relevant to its distinct demography, but body size alone cannot distinguish heritable local adaptation from ecological or demographic effects. We consequently do not exclude incipient subspecies formation; however, the present genome-wide data show low absolute divergence and no deeply separated nuclear lineage, so formal subspecies recognition would require additional evidence for persistent adaptive or morphological differentiation and reproductive isolation. The increased ROH and reduced diversity also identify a biologically plausible risk of inbreeding depression if effective population size declines further.

The sperm whale presents a striking example of a global effective panmixia, as there is no meaningful population differentiation across all oceans that can be recovered in different approaches, except for some differentiation of the Mediterranean population [15]. The theoretical concept and theoretical ideal of panmixia, that of identical probability of reproducing, is unrealistic under natural conditions, where geographic distances, social structures, or behavioral differences naturally limit mating opportunities. Nevertheless, in effective panmixia, frequent gene flow between individuals over large distances can approximate the theoretical concept, leading to and maintaining a shared genepool, homogenizing allele frequencies across space, which offers a more general and realistic definition of panmixia.

Natural examples of panmixia are rare and typically involve species with relatively limited geographic ranges, most of which are marine: the European eel, *Anguilla anguilla* [91,92,93], the blue hake, *Antimora rostrata* [7], the goby, *Caffrogobius caffer* [94], and the skipjack tuna *Katsuwonus pelamis* [95]. These cases illustrate that strictly equal mating probabilities are not always considered necessary for, in effective, panmictic population structure. Instead, factors such as high dispersal capacity, strong habitat connectivity, and ecological pressures that promote gene flow can result in populations that are effectively panmictic at the genetic level, even if individual-level mating remains non-random.

Panmixia has significant biological implications for genetic diversity and evolutionary dynamics. It maintains a greater heterozygosity, and thus resilience to diseases or environmental change [96]. Inbreeding depression, which can occur in isolated or bottlenecked populations, can be expected to be low in large panmictic populations. In a large, panmictic population (species), genetic loss due to drift is reduced too, due to the larger effective population size Ne [97].

The genomic homogeneity, high heterozygosity, limited ROHs in sperm whales, and panmixia aid global conservation efforts. Previous studies have suggested a relatively low genetic diversity of sperm whales compared to other large cetaceans [15,26] and raised concerns about the consequences of the intensive commercial whaling in the past. While the genetic diversity before commercial whaling is not known, observed heterozygosity of ~0.12% falls within the range for other wildlife [98], and for mammals is found to range between 0.02 and 0.3%. In the sperm whale, heterozygosity is higher than that of the polar bear (~0.05%) [99]. Inbreeding, as indicated by runs of homozygosity, seems to be a sporadic sampling artifact and is likely a rare consequence of the rather stationary behavior of females [27].

Although this study does not include samples from Australia, Asia, or the Southern Ocean, earlier research has not found distinct populations in these areas [22], unlike, for example, the pygmy blue whale [3,23]. Therefore, it is unlikely that significant hidden differences exist in these regions.

In general, high connectivity as in panmixia counteracts local adaptation, slows the pace of divergence, and buffers populations from genetic drift [9,100]. In such systems, heterozygosity and overall genetic diversity are typically maintained, as allele loss due to drift is minimized and no isolated pockets are subject to independent evolutionary trajectories. As a result, evolutionary change is slower, and populations remain genetically cohesive across broad ranges [101], thus working against population structuring, subspecies formation, and, subsequently, speciation.

## 5. Conclusions

Genome-wide analyses of 45 sperm whales support effective panmixia across the sampled Atlantic, Indian, and Pacific regions, with little consistent geographic structure across nuclear phylogenies, PCoA, admixture, genetic-distance, and demographic analyses. The Mediterranean population is the main exception, showing reduced heterozygosity, increased runs of homozygosity, and moderately elevated, consistent with restricted gene flow, reduced effective population size, and stronger genetic drift. These results indicate demographic differentiation rather than clear taxonomic separation and emphasize the conservation importance of Mediterranean sperm whales.

The main limitation is uneven geographic sampling, particularly the small sample sizes from the East Pacific and Indian Ocean and the absence of samples from several major regions. Therefore, weak regional structure or local adaptation cannot be excluded. Future studies should include larger, more balanced samples, both sexes, and multiple social groups, together with high-coverage genomic, movement, and acoustic data. More specifically, future sampling should extend into the Arctic and adjacent high-latitude regions, where recent records indicate high-latitude occurrence and long-range migration [73,74,75], and should include a larger proportion of females from multiple social units. This would strengthen direct tests of the geographic scale of female philopatry, sex-biased dispersal, local adaptation, and whether regional differentiation such as that observed in the Mediterranean reflects stable demographic isolation or incipient evolutionary divergence.

## Data Availability

The sequencing data generated in this study have been deposited in the NCBI Sequence Read Archive (SRA) under BioProject accession PRJNA1305154.

## Supplementary Materials

The following supporting information can be downloaded at https://www.mdpi.com/article/10.3390/biology15181619/s1, Table S1. General information of 41 sperm whales sequenced in this study. Provided are field-IDs, names, location, and sex. Table S2. General information for samples retrieved for the population genetic analyses from the NCBI sequence read archive (SRA). Samples are sorted by the Bioproject number. Provided are the species name and the respective study, the sample ID provided by the submitting authors, Biosample and SRA accession numbers, as well as known location and sampling years if available. Table S3. Mapping stats summary of the samples used in the study. Table S4. Mean depth of the samples calculated from a thinned dataset. Table S5. Kinship analysis (Excel sheet). Supplementary Figure S1. Autosomal depth filter. Each dot represents the sequencing for all samples combined for a single site, along a randomly chosen autosome. The red lines indicate the minimum and maximum depth filter threshold used when filtering the data, i.e., 225 and 700. For a dataset of 45 individuals, and assuming a mean autosomal sequencing depth of 10×, the expected combined depth is 45 × 10 = 450. Because four individuals were sequenced with a mean depth of 20×, the actual overall mean value is a bit higher. Supplementary Figure S2. Autosomal depth filter. Histogram of combined depth per site. The red lines indicate the minimum and maximum depth filter threshold used when filtering the data along with percentage of retained and discarded number of sites. Supplementary Figure S3A and B. X-chromosomal depth filter. Left: Each dot represents the sequencing for all samples combined for a single site from a random subregion of the X-chromosome. The red lines indicate the minimum and maximum depth filter threshold used when filtering the data, i.e., 200 and 500. Right: Histogram of combined depth per site. The red lines indicate the minimum and maximum depth filter threshold used when filtering the data. Supplementary Figure S4. Heatmap depicting Dxy estimates between individuals, in percentages. Genetic clusters are inferred using arbitrary threshold values between 0.10% and 0.12%. At this and other distance thresholds, no obvious clustering/grouping can be observed that does not show any population structure. Supplementary Figure S5. Population-pairwise Dxy-estimates. Mean absolute genetic distances between pairs of populations. Note the marginal differences between populations that do not show any population structure. Supplementary Figure S6. Genetic structure of sperm whale populations based on autosomal genes: Unrooted phylogenetic network constructed using the NeighborNet algorithm and based on pairwise autosomal sequence dissimilarities calculated from a thinned set of polymorphic markers. The network shows a clear separation of the Mediterranean population, with all other populations forming a densely interconnected network in a single node, suggesting panmixia. Supplementary Figure S7. Genetic structure of sperm whale populations based on autosomal markers including an outgroup: Genetic structure of sperm whales based on autosomal sequence dissimilarity using the BioNJ algorithm. The phylogeny was constructed with *Kogia breviceps* (pygmy sperm whale) included as an outgroup. The resulting topology displays a characteristic sunflower-like shape, reflecting the deep divergence between *K. breviceps* and *Physeter macrocephalus*. As expected, an outgroup does not help to clarify the unresolved sperm whale dendrogram. Supplementary Figure S8. Genetic structure of sperm whale populations based on autosomal markers in a rectangular shape: Rooted nuclear phylogeny of sperm whales using *Kogia breviceps* (pygmy sperm whale) as an outgroup. The tree was generated from autosomal sequence dissimilarity using the BioNJ algorithm and shows no apparent genetic structure among global sperm whale individuals, consistent with panmixia. The long branch separating *K. breviceps* from *Physeter macrocephalus* reflects their deep evolutionary divergence, while the shallow internal branches among sperm whales indicate low genomic differentiation across geographic regions. The rectangular shape of the tree does not aid in depicting higher population order by geography that might be hidden in the circular shape. All terminal branches end in almost non-existing internal branches. Supplementary Figure S9. Neighbor-joining phylogram of all sperm whale individuals based on Rogers genetic distance, constructed using the poppr package in R. Bootstrap values (BV) are based on 1000 replicates, and only nodes with BV > 50% are shown. The absence of well-supported clusters, including for the Mediterranean individuals (indicated by star shape < 50% BV), highlights weak phylogeographic structure and overall panmixia. Supplementary Figure S10. Treemix analysis showing very short internal branches consistent with the absence of population structure. Supplementary Figure S11. Rooted phylogram based on mitochondrial genome sequences for TMRCA analysis, constructed using IQ-TREE. Populations are color-coded, and the limited node support values are shown. The tree highlights some matrilineal signal patterns, with some clustering but not by geographic region. Supplementary Figure S12. A,B. Validation of run of homozygosity analyses. Line charts depicting fluctuations of heterozygosity levels (He) across two randomly chosen chromosomes. Grey segments highlight the genomic regions which are marked as run of homozygosity (ROH) by the software Darwindow. Values along the y-axis represent sample- and chromosome-specific FROH-values, the proportion of the chromosome marked as ROH. White regions are excluded from the analyses owing to the high proportions of missing data. Supplementary Figure S13. Principal coordinate analysis (PCoA) after excluding the inbred individuals GOM5, NORTHSEA3, and NORTHSEA6, showing no detectable population structure in sperm whales. Supplementary Figure S14. Population-pairwise Hudson Fst-estimates. Mean relative genetic distances between pairs of populations. Supplementary Figure S15. Population-pairwise Weir and Cockerham’s Fst-estimates. Warmer colors and higher Fst values indicate greater genetic differentiation between populations. The Mediterranean population shows notably higher differentiation compared to other North Atlantic populations. Supplementary Figure S16. Pairwise Fst *p*-values calculated using the STAMPP package in R with 500 bootstrap replicates. Statistically significant *p*-values (*p* < 0.05) indicate genetic differentiation between population pairs. The Mediterranean population is significantly different from North Atlantic populations, as reflected by the *p*-values (0) along the corresponding matrix rows and columns. Supplementary Figure S17. Demographic analyses using stairway plots calculated using a mutation rate 2.5 × 10^−8^ and generation time of 30 years. Red line indicates the median values. Black lines indicate 12.5% and 87.5% percentile values, and grey margins indicate 2.5% and 97.5% percentile values. Supplementary Figure S18. Unrooted mitochondrial phylogeny of sperm whales based on the integrated mtDNA dataset using IQ-TREE. Unrooted phylogenetic tree reconstructed from mitochondrial genome sequences, including all of the published mitogenomic haplotypes and the mitochondrial genomes extracted from the present study. Tips are color-coded by sampling region. The tree shows mixed clustering of haplotypes across ocean basins, with no clean ocean-specific clades. This pattern indicates limited matrilineal structure but does not support strong ocean-basin subdivision across the global dataset. Bootstrap support values are shown at major nodes; branch lengths are proportional to genetic distance. Supplementary Figure S19. Rooted mitochondrial maximum likelihood phylogeny based on the integrated mtDNA dataset. Rooted mitochondrial phylogeny reconstructed from the same integrated mtDNA dataset and linearized to estimate relative divergence times among haplotypes. Tips are color-coded by sampling region, and node labels indicate bootstrap support.
