# Supplementary material for: Genome Analyses Support Global Effective Panmixia in Sperm Whales
Source: Biology (Basel). 2026 Sep 14;15(18):1619. doi: 10.3390/biology15181619 (PMC13604797; doi:10.3390/biology15181619)
Supplement: Supplementary file 1 [file biology-15-01619-s001.zip › Supplementary_Tables_and_Figures-MBA-v20.pdf]

Table S1 General information of 41 sperm whales sequenced in this study. Provided are field-IDs , names, location and sex.

Species	ID	Name	Sex	Location
SpermWhale	PC1	Med1	M	Mediterranean
SpermWhale	PC3	Med2	M	Mediterranean
SpermWhale	SW275B	Med3	M	Mediterranean
SpermWhale	F35	Med5	M	Mediterranean
SpermWhale	F71	Med6	M	Mediterranean
SpermWhale	F53	Med7	M	Mediterranean
SpermWhale	M143962	NorthSea1	M	NorthSea
SpermWhale	M143963	NorthSea2	M	NorthSea
SpermWhale	M143964	NorthSea3	M	NorthSea
SpermWhale	M143965	NorthSea4	M	NorthSea
SpermWhale	M143966	NorthSea5	M	NorthSea
SpermWhale	M2583947	NorthSea6	M	NorthSea
SpermWhale	M2583948	NorthSea7	M	NorthSea
SpermWhale	M2583949	NorthSea8	M	NorthSea
SpermWhale	70201	GOM1	F	Gulf_of_Mexico
SpermWhale	1040101	GOM2	M	Gulf_of_Mexico
SpermWhale	1040102	GOM3	M	Gulf_of_Mexico
SpermWhale	71301	GOM4	M	Gulf_of_Mexico
SpermWhale	71601	GOM5	M	Gulf_of_Mexico
SpermWhale	72604	GOM6	M	Gulf_of_Mexico
SpermWhale	1031704	GOM7	M	Gulf_of_Mexico
SpermWhale	72601	GOM8	M	Gulf_of_Mexico
SpermWhale	A155	Azores1	M	Azores
SpermWhale	A192	Azores2	M	Azores
SpermWhale	A223	Azores3	F	Azores
SpermWhale	M1	Madeira1	M	Madeira
SpermWhale	M4	Madeira2	M	Madeira
SpermWhale	M14	Madeira3	F	Madeira
SpermWhale	C10	Canary1	M	CanaryIslands
SpermWhale	C15	Canary2	M	CanaryIslands
SpermWhale	NS2	Galicia1	M	Galicia
SpermWhale	NS5	Galicia3	F	Galicia
SpermWhale	F22	ICELAND1	M	ICELAND
SpermWhale	F27	ICELAND2	M	ICELAND
SpermWhale	F31	ICELAND3	M	ICELAND
SpermWhale	F33	ICELAND4	M	ICELAND

SpermWhale	F39	ICELAND6	M	ICELAND
SpermWhale	F40	ICELAND7	M	ICELAND
SpermWhale	F41	ICELAND8	M	ICELAND
SpermWhale	F43	ICELAND9	M	ICELAND
SpermWhale	F45	ICELAND10	M	ICELAND

Table S2 General information for samples retrieved for the population genetic analyses from the NCBI sequence read archive (SRA). Samples are sorted by the BioProject. Provided are the species name and the respective study, the sample ID provided by the submitting authors, Biosample and SRA accession numbers as well as known location and sampling years if available

Source	ID	Biosample/SRA accession	Bioproject	Name	Sex	Study
NCBI	SC-2-Pacific	SRX2447269, SRX2447272	PRJNA89089;	EastPacific1	F	Warren et al. 2017, The Novel Evolution of the Sperm Whale Genome
NCBI	SC-1-Pacific	SRX2447271, SRX2447274	PRJNA89089	EastPacific2	F	Warren et al. 2017, The Novel Evolution of the Sperm Whale Genome
NCBI	SEY-2-Indian	SRX2447270, SRX2447273	PRJNA89089	IndianOcean1	F	Warren et al. 2017, The Novel Evolution of the Sperm Whale Genome
NCBI	SEY-1-Indian	SRX2447268, SRX2447275	PRJNA89089	IndianOcean2	F	Warren et al. 2017, The Novel Evolution of the Sperm Whale Genome

Table S3 Mapping stats summary of the samples used in the study

**Name**	**Mapped%**	**Properly paired %**
AZORES1	99.74%	93.87%
AZORES2	99.68%	89.92%
AZORES3	99.82%	94.05%
CANARY1	98.49%	92.68%
CANARY2	98.02%	90.42%
EastPacific1	99.50%	92.62%
EastPacific2	99.38%	92.47%
GALICIA1	99.50%	86.91%
GALICIA3	99.73%	94.03%
GOM1	99.77%	93.34%
GOM2	99.73%	93.14%
GOM3	99.71%	93.25%
GOM4	99.72%	93.66%
GOM5	99.64%	92.51%
GOM6	99.69%	93.31%
GOM7	99.76%	92.33%
GOM8	99.76%	92.32%
ICELAND10	99.70%	93.07%
ICELAND1	99.69%	91.95%
ICELAND2	99.74%	92.97%
ICELAND3	99.63%	92.29%
ICELAND4	99.72%	92.94%
ICELAND6	99.67%	93.56%
ICELAND7	99.62%	91.85%
ICELAND8	99.61%	92.87%
ICELAND9	99.69%	93.13%
IndianOcean1	98.81%	90.19%
IndianOcean2	99.49%	77.17%
MADEIRA1	99.70%	93.20%
MADEIRA2	99.68%	93.79%
MADEIRA3	99.82%	94.59%
MED1	99.69%	93.38%
MED2	99.66%	92.39%
MED3	99.50%	92.44%
MED5	99.71%	93.53%

MED6	99.73%	94.11%
MED7	99.74%	93.26%
NORTHSEA1	99.73%	92.29%
NORTHSEA2	99.77%	92.93%
NORTHSEA3	99.36%	93.00%
NORTHSEA4	99.75%	94.09%
NORTHSEA5	99.78%	94.10%
NORTHSEA6	99.67%	92.97%
NORTHSEA7	99.61%	93.46%
NORTHSEA8	99.72%	93.40%

Table S4 Mean depth of the samples calculated from thinned dataset

**INDV**	**MEAN_DEPTH**
AZORES1	12.043
AZORES2	8.98608
AZORES3	8.38472
CANARY1	8.55942
CANARY2	9.65345
EastPacific1	18.8343
EastPacific2	22.1896
GALICIA1	8.32062
GALICIA3	9.96347
GOM1	10.8248
GOM2	10.7449
GOM3	8.15993
GOM4	7.68781
GOM5	7.81548
GOM6	7.50986
GOM7	8.65418
GOM8	8.98877
ICELAND10	8.10916
ICELAND1	7.35054
ICELAND2	7.56061
ICELAND3	7.15006
ICELAND4	7.95221
ICELAND6	6.34933
ICELAND7	6.42602
ICELAND8	7.12053

ICELAND9	23.8528
IndianOcean1	17.9798
IndianOcean2	12.0965
MADEIRA1	8.80862
MADEIRA2	9.03674
MADEIRA3	8.93211
MED1	9.4249
MED2	8.0268
MED3	10.3486
MED5	10.5406
MED6	9.49397
MED7	9.5705
NORTHSEA1	8.83893
NORTHSEA2	9.22112
NORTHSEA3	9.52651
NORTHSEA4	10.3525
NORTHSEA5	9.71051
NORTHSEA6	9.27755
NORTHSEA7	8.80563
NORTHSEA8	10.4761

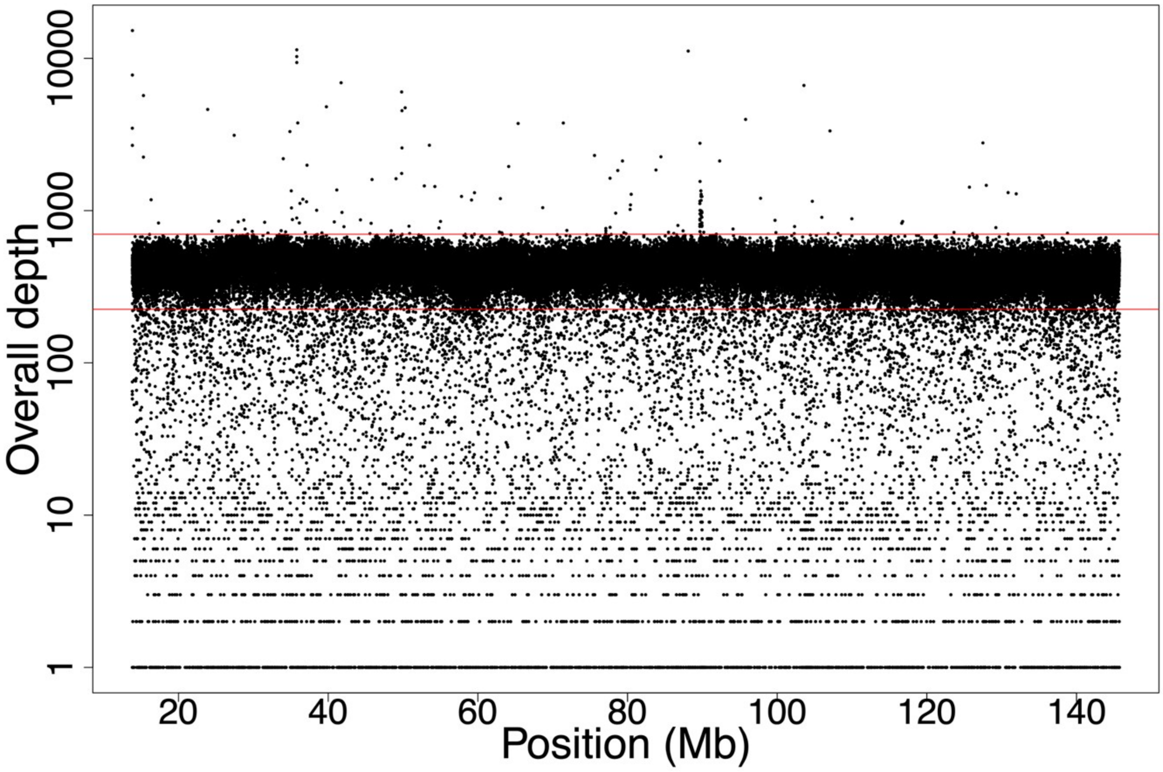

Supplementary Figure S1. **Autosomal depth filter**. Each dot represents the sequencing for all samples combined for a single site, along a randomly chosen autosome. The red lines indicate the minimum and maximum depth filter threshold used when filtering the data i.e. 225 and 700. For a dataset of 45 individuals, and assuming a mean autosomal sequencing depth of 10x, the expected combined depth is 45*10 = 450. Because four individuals were sequenced with a mean depth of 20x, the actual overall mean value is a bit higher.

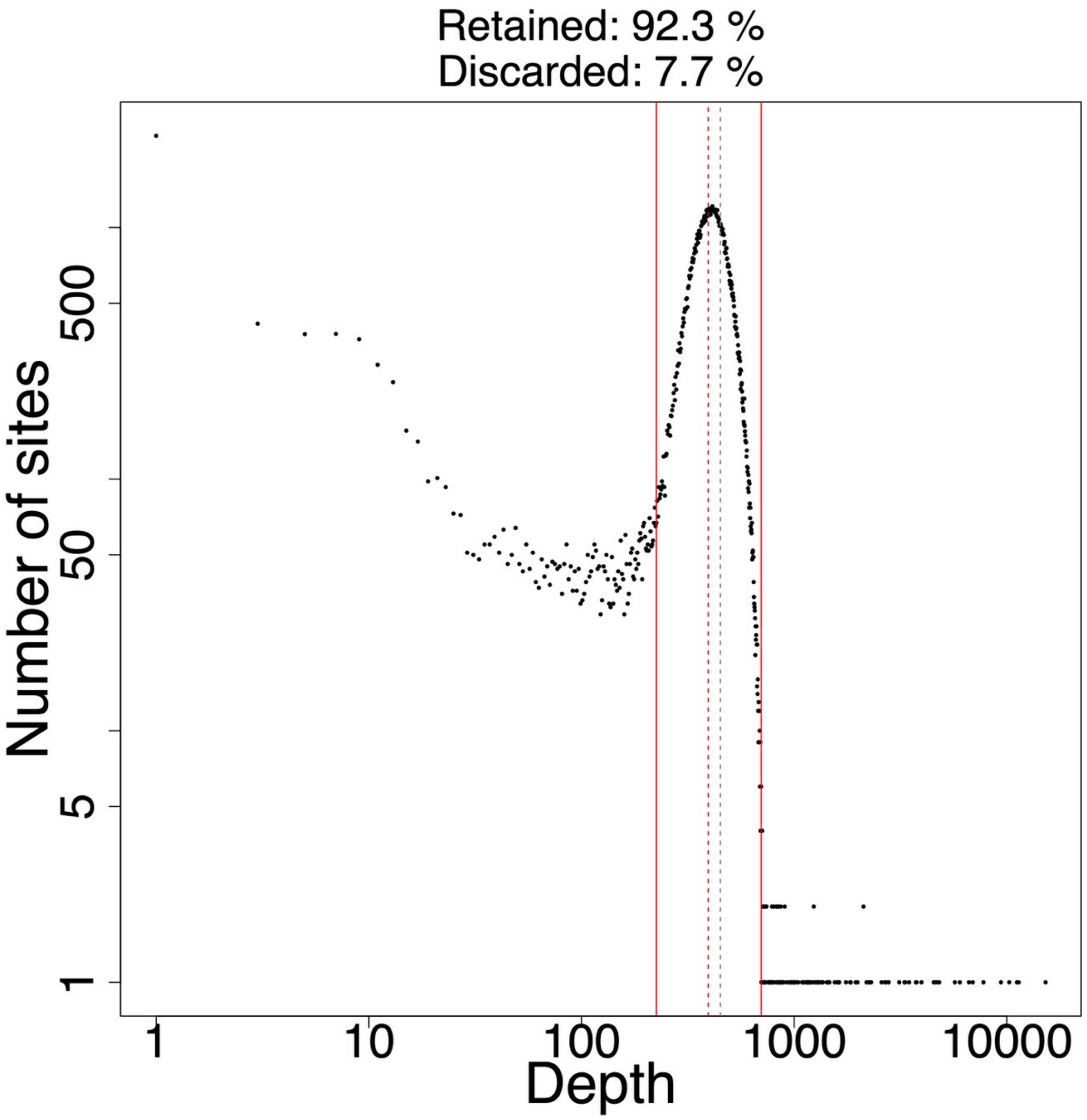

Supplementary Figure S2. **Autosomal depth filter** Histogram of combined depth per site. The red lines indicate the minimum and maximum depth filter threshold used when filtering the data along with percentage of retained and discarded number of sites.

A

B

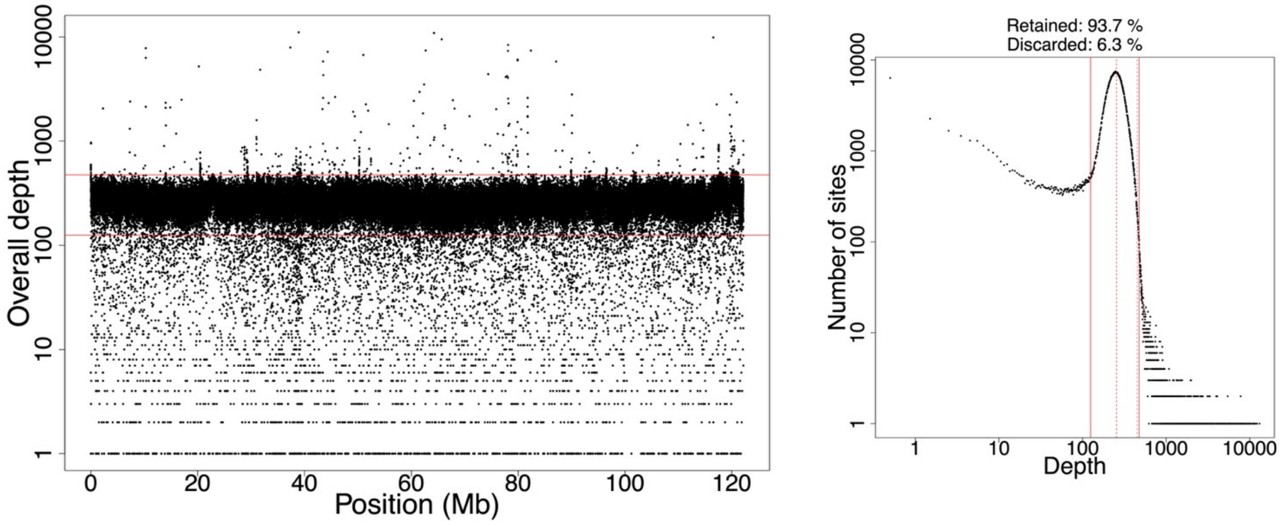

Supplementary Figure S3A and B. ***X-chromosomal depth filter. Left***: Each dot represents the sequencing for all samples combined for a single site from a random subregion of the X-chromosome. The red lines indicate the minimum and maximum depth filter threshold used when filtering the data i.e. 200 and 500. ***Right***: Histogram of combined depth per site. The red lines indicate the minimum and maximum depth filter threshold used when filtering the data.

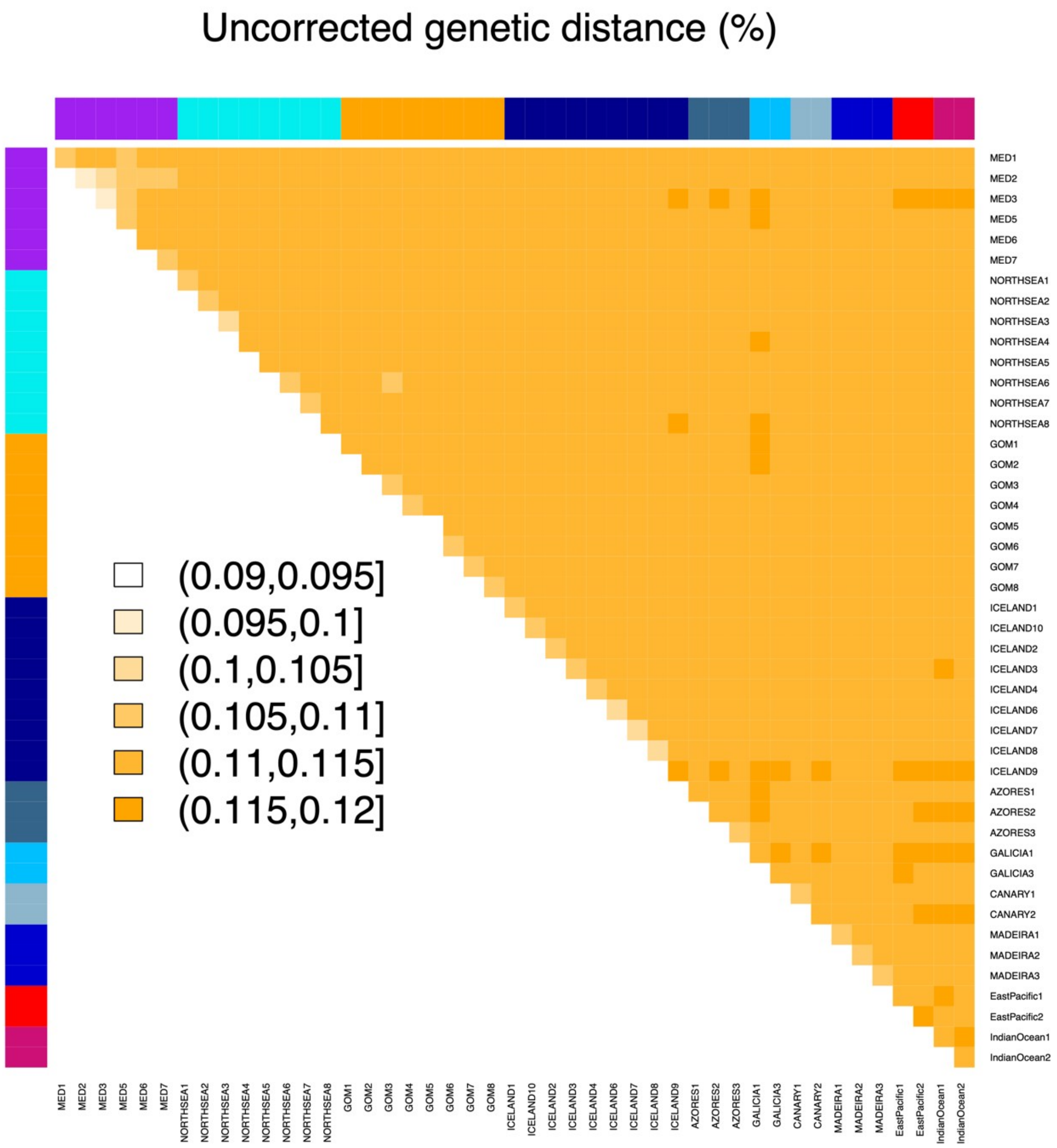

Supplemental Figure S4. Heatmap depicting Dxy estimates between individuals, in percentages. Genetic clusters are inferred using arbitrary threshold values between 0.10% and 0.12%. At this and other distance threshold no obvious clustering/grouping can be observed that do not show any population structure.

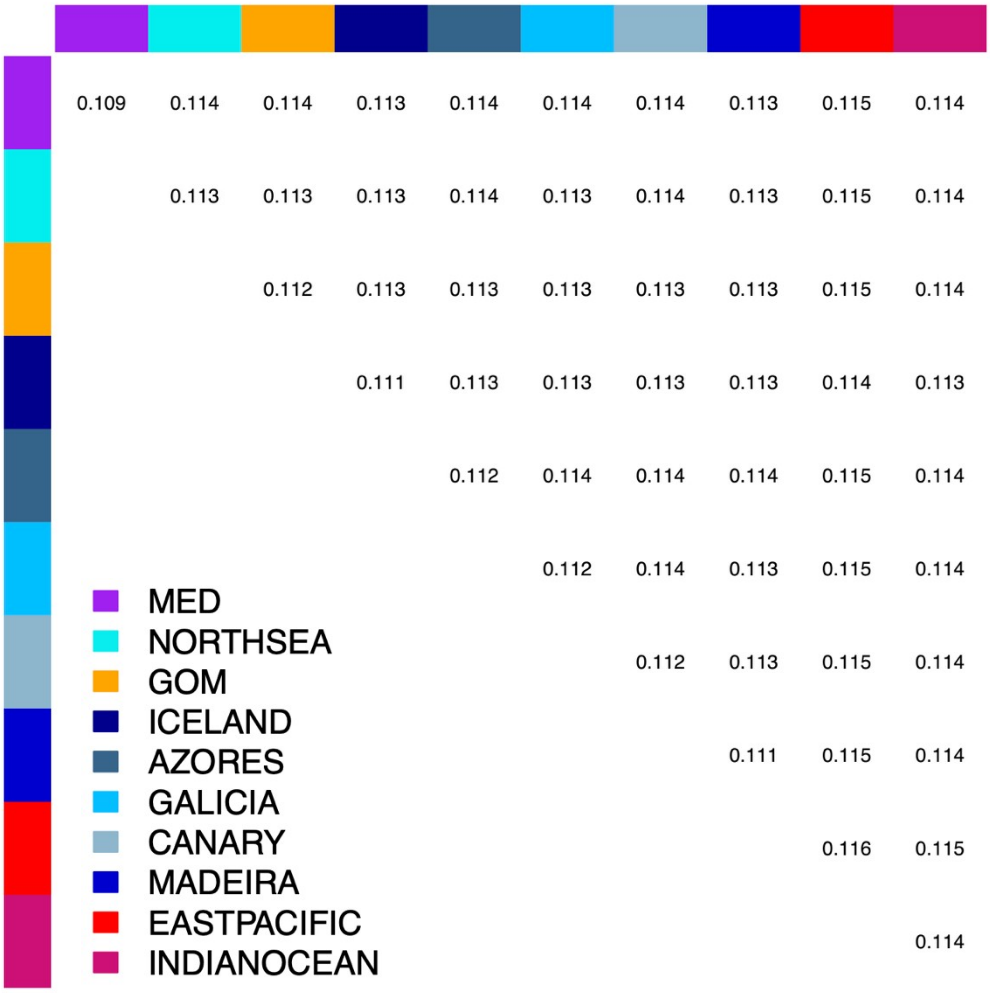

Supplemental Figure S5. Population-pairwise Dxy-estimates. Mean absolute genetic distances between pairs of populations. Note the marginal differences between populations that do not show any population structure.

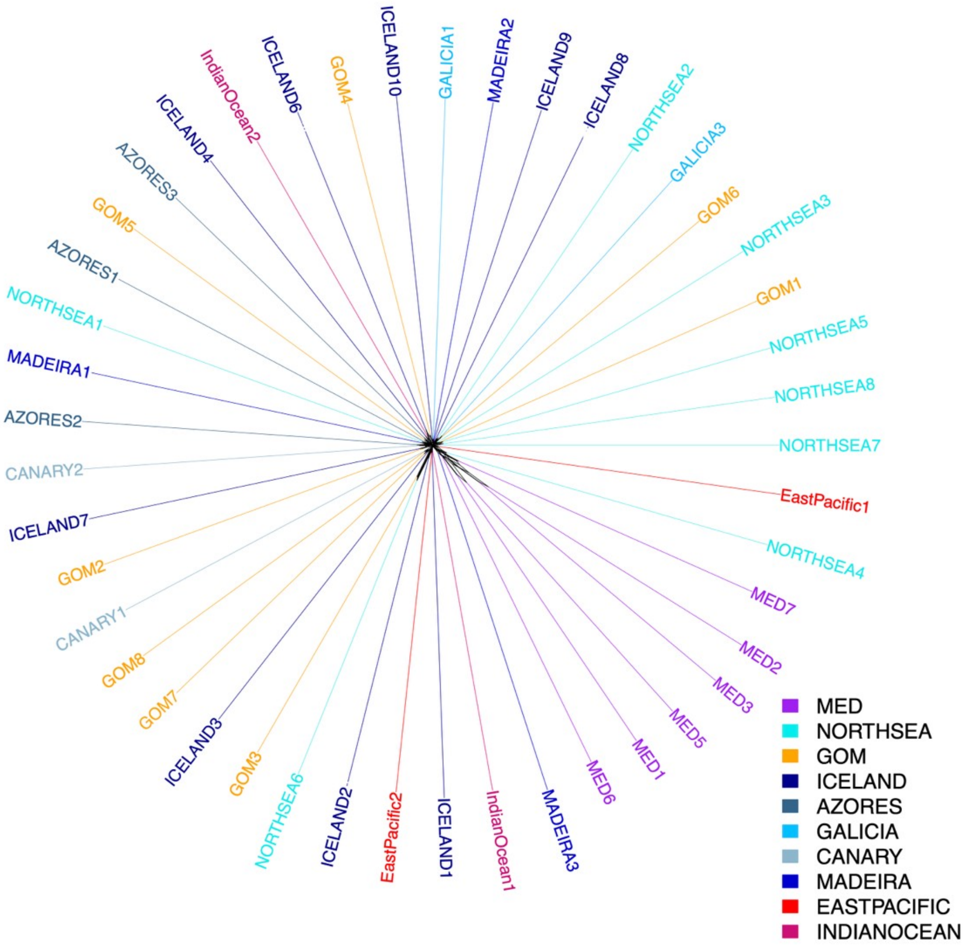

Supplementary Figure S6: Genetic structure of sperm whale populations based on autosomal: Unrooted phylogenetic network constructed using the NeighborNet algorithm and based on pairwise autosomal sequence dissimilarities calculated from a thinned set of polymorphic markers. The network shows a separation of the Mediterranean population, with all other populations forming a densely interconnected in a single node, suggesting panmixia.

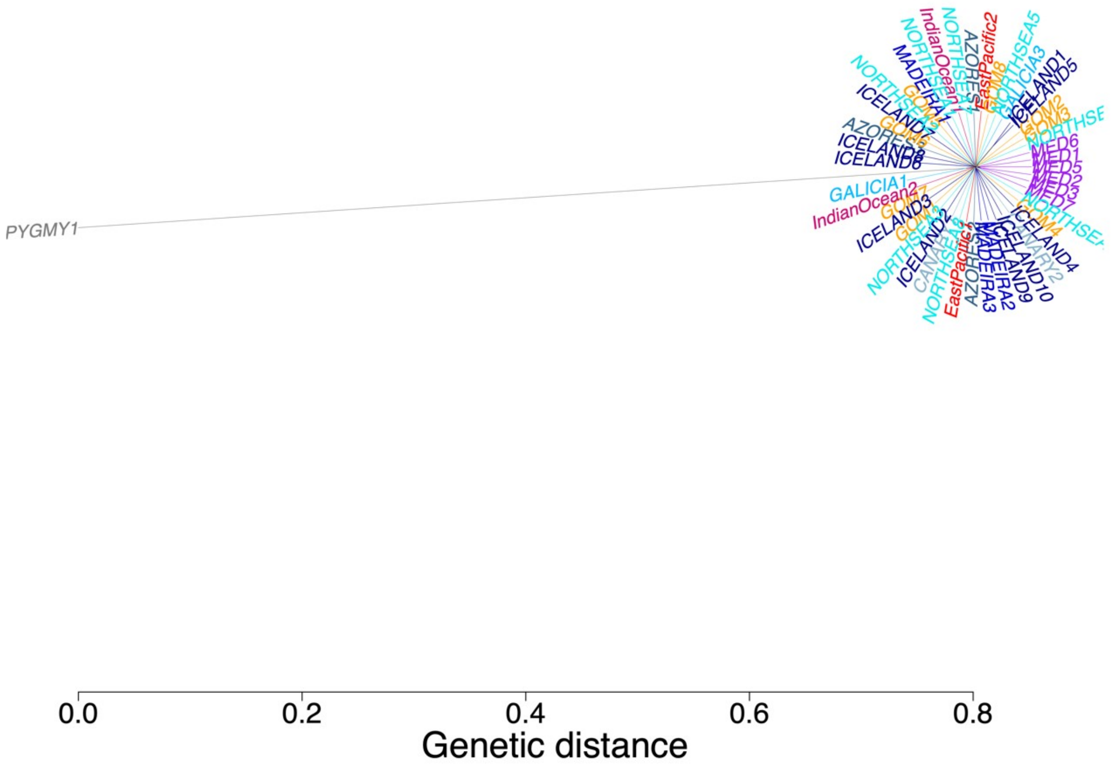

Supplementary Figure S7: Genetic structure of sperm whale populations based on autosomal markers including an outgourp: Genetic structure of sperm whales based on autosomal sequence dissimilarity using the BioNJ algorithm. The phylogeny was constructed with *Kogia breviceps* (pygmy sperm whale) included as an outgroup. The resulting topology displays a characteristic sunflower-like shape, reflecting the deep divergence between *K. breviceps* and *Physeter macrocephalus*. As expected, an outgroup does not help to clarify the unresolved sperm whale dendrogram.

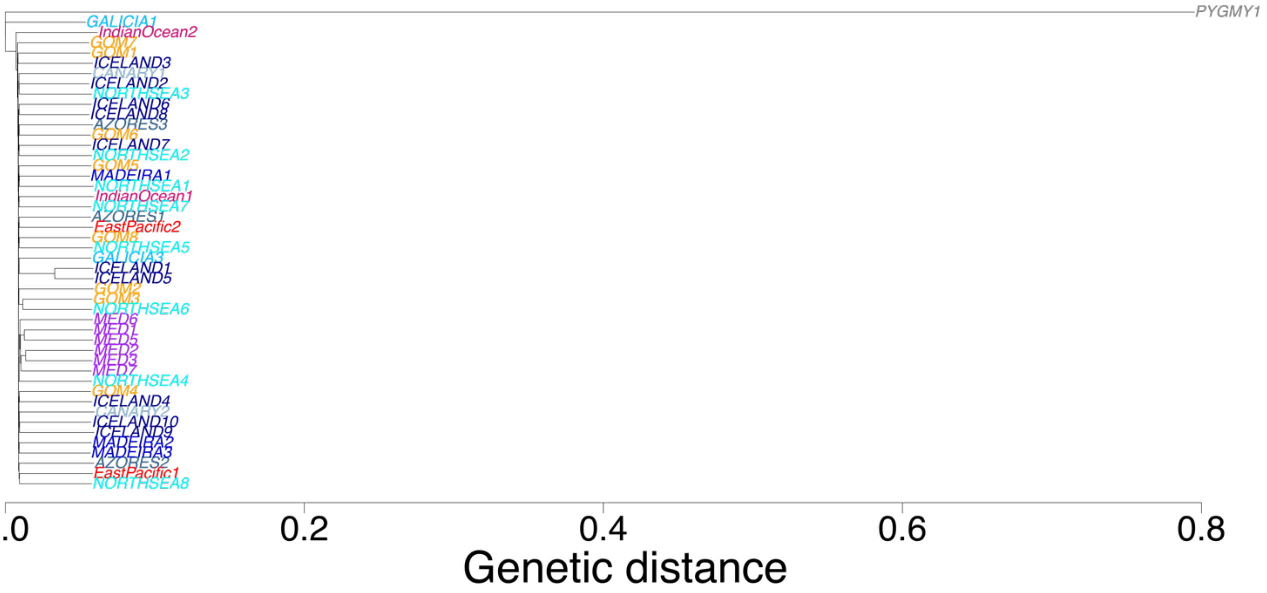

Supplementary Figure S8: Genetic structure of sperm whale populations based on autosomal markers in a rectangular shape: Rooted nuclear phylogeny of sperm whales using *Kogia breviceps* (pygmy sperm whale) as an outgroup. The tree was generated from autosomal sequence dissimilarity using the BioNJ algorithm and shows no apparent genetic structure among global sperm whale individuals, consistent with panmixia. The long branch separating *K. breviceps* from *Physeter macrocephalus* reflects their deep evolutionary divergence, while the shallow internal branches among sperm whales indicate low genomic differentiation across geographic regions. The recangular shape of the tree does not aid in depicting higher population order by geography that might be hidden in the circular shape. All terminal branches end in almost non exisiting internal branches.

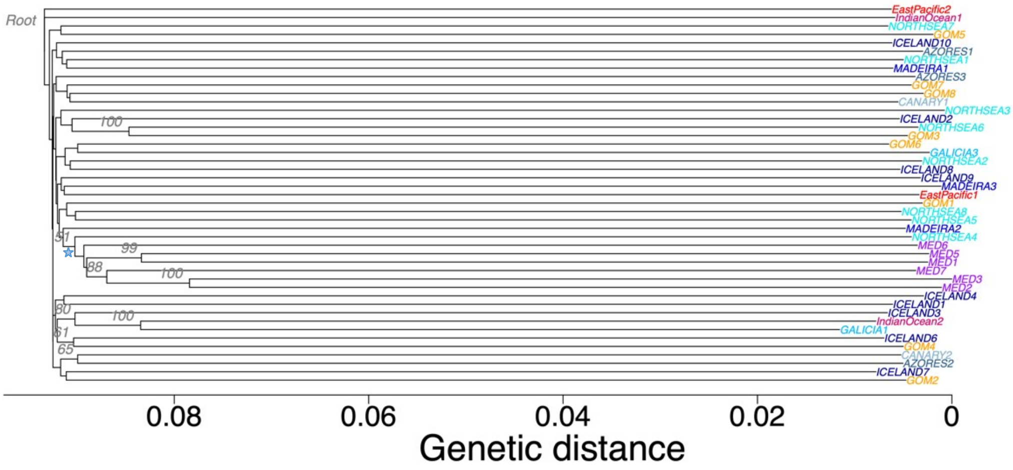

Supplementary Figure S9: Neighbor-joining phylogram of all sperm whale individuals based on Rogers genetic distance, constructed using the poppr package in R. Bootstrap values (BV) are based on 1000 replicates, and only nodes with BV > 50% are shown. The absence of well-supported clusters, including for the Mediterranean individuals (indicated by star shape <50% BV), highlights weak phylogeographic structure and overall panmixia

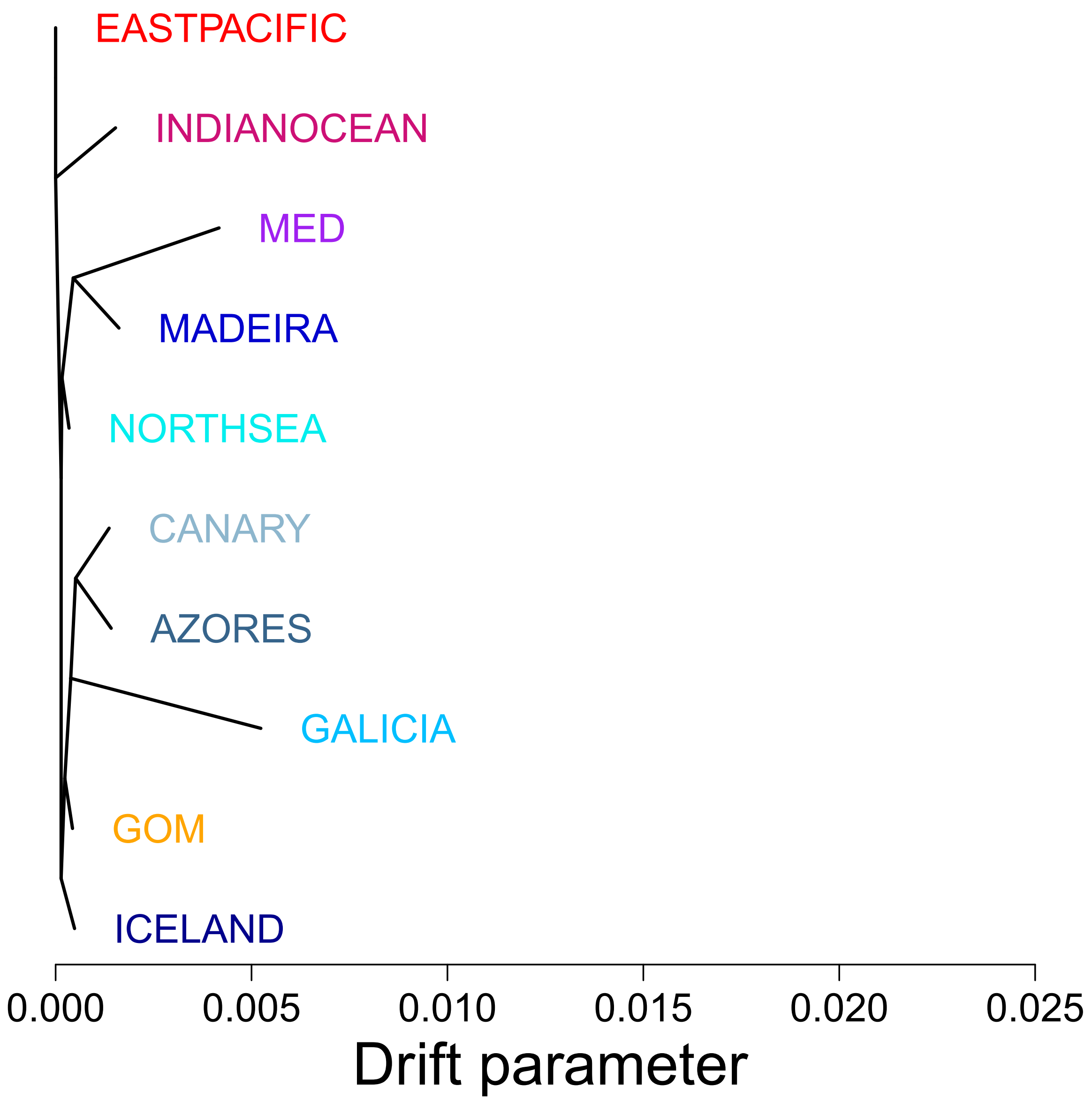

Supplementary Figure S10. Treemix analysis showing very short internal branches consistent with the absence of population structure.

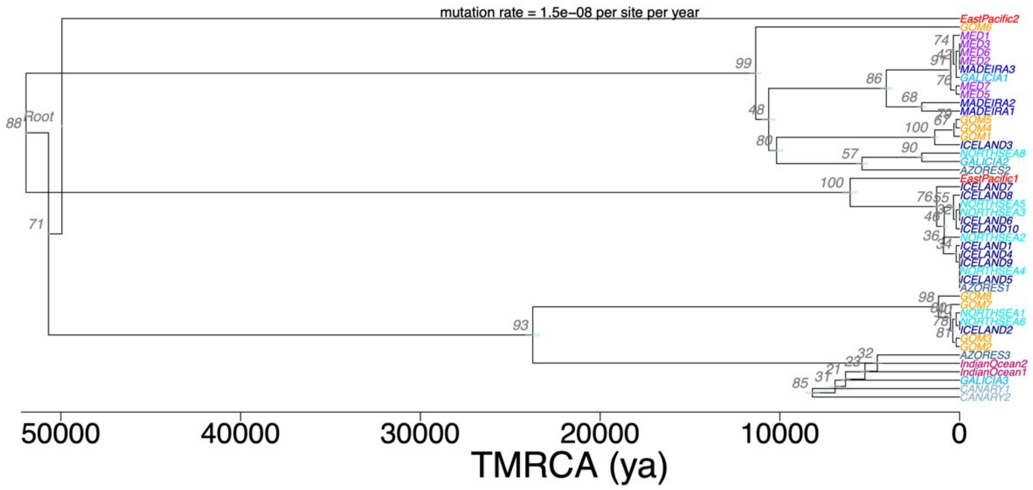

Figure S11: Rooted phylogram based on mitochondrial genome sequences for TMRCA analysis, constructed using IQ-TREE. Populations are color-coded, and the limited node support values are shown. The tree highlights some matrilineal signal patterns, with some clustering but not by geographic region.

A

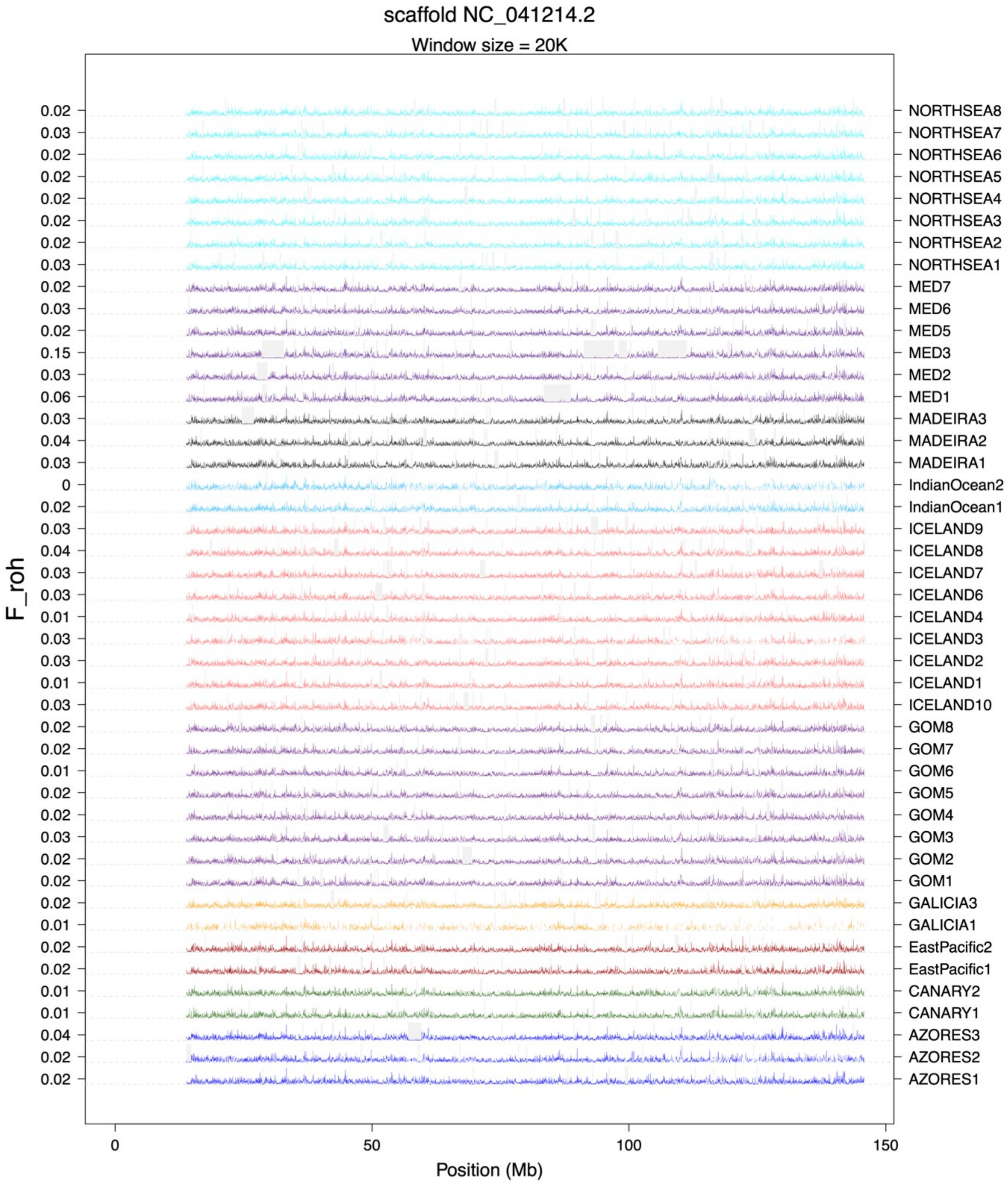
scaffold NC_041214.2
Window size = 20K
F_roh
Position (Mb)
0
50
100
150
0.02 NORTHSEA8
0.03 NORTHSEA7
0.02 NORTHSEA6
0.02 NORTHSEA5
0.02 NORTHSEA4
0.02 NORTHSEA3
0.02 NORTHSEA2
0.03 NORTHSEA1
0.02 MED7
0.03 MED6
0.02 MED5
0.15 MED3
0.03 MED2
0.06 MED1
0.03 MADEIRA3
0.04 MADEIRA2
0.03 MADEIRA1
0 IndianOcean2
0.02 IndianOcean1
0.03 ICELAND9
0.04 ICELAND8
0.03 ICELAND7
0.03 ICELAND6
0.01 ICELAND4
0.03 ICELAND3
0.03 ICELAND2
0.01 ICELAND1
0.03 ICELAND10
0.02 GOM8
0.02 GOM7
0.01 GOM6
0.02 GOM5
0.02 GOM4
0.03 GOM3
0.02 GOM2
0.02 GOM1
0.02 GALICIA3
0.01 GALICIA1
0.02 EastPacific2
0.02 EastPacific1
0.01 CANARY2
0.01 CANARY1
0.04 AZORES3
0.02 AZORES2
0.02 AZORES1

B

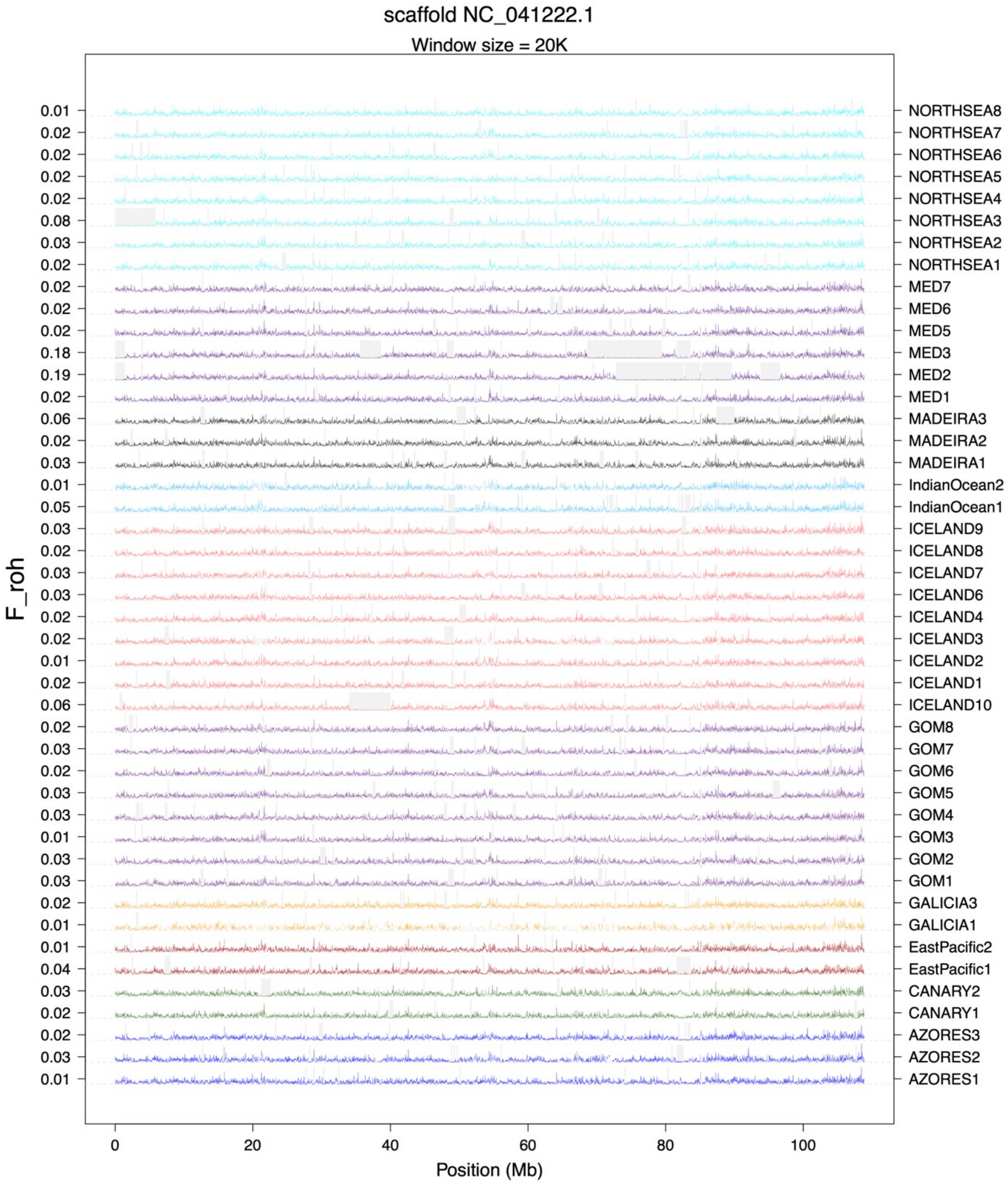

Supplementary Figure S12. (A,B) Validation of run of homozygosity analyses. Line charts depicting fluctuations of heterozygosity levels (He) across two randomly chosen chromosomes. Grey segments highlight the genomic regions which are marked as run of homozygosity (ROH) by the software Darwindow. Values along the y-axis represent sample and chromosome-specific FROH-values, the proportion of the chromosome marked as ROH. White regions are excluded from the analyses owing the high proportions of missing data.

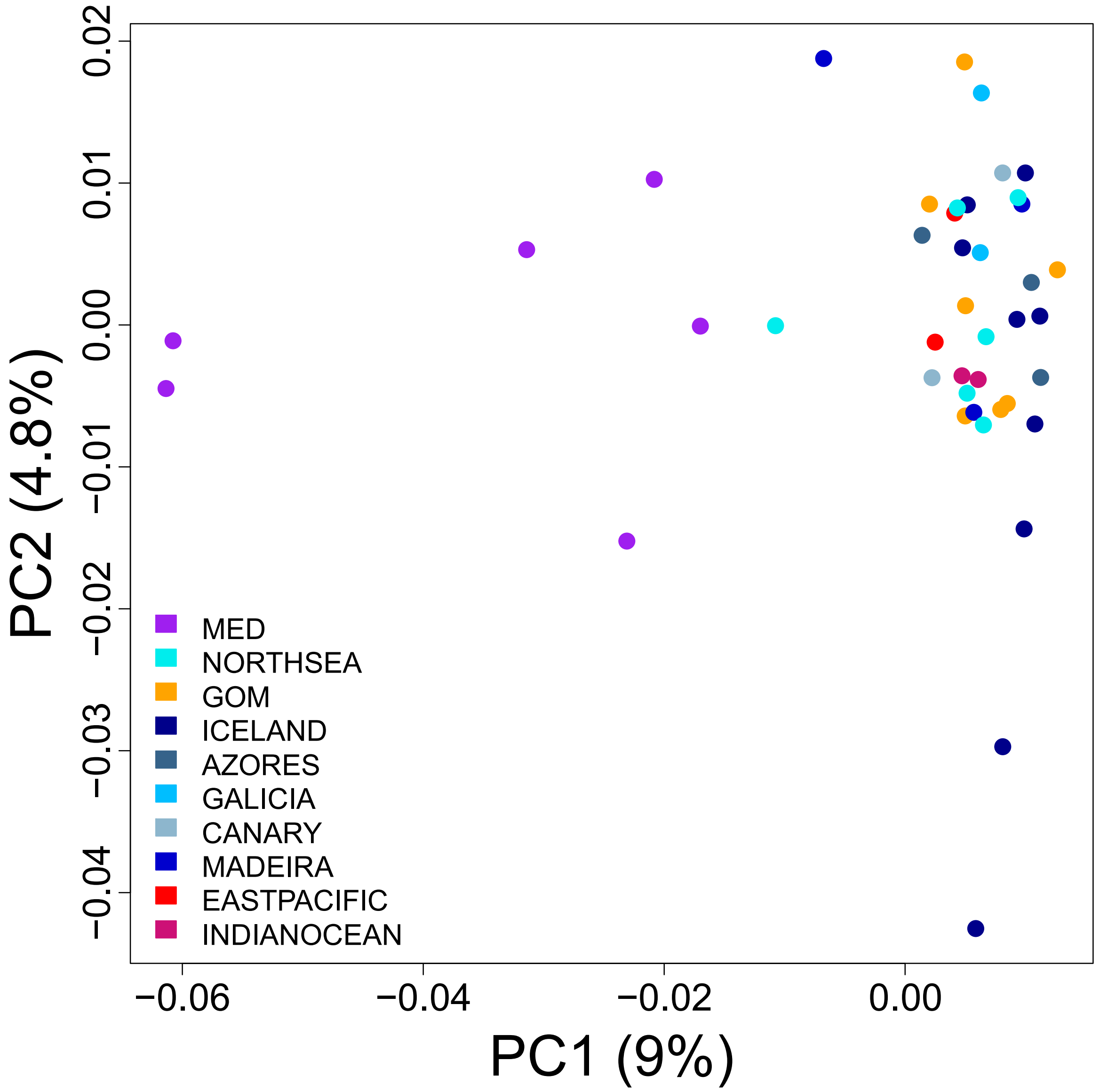

Supplementary Figure S13: Principal coordinate analysis (PCoA) after excluding the inbred individuals GOM5, NORTHSEA3, and NORTHSEA6, showing no detectable population structure in sperm whales.

	0.026	0.03	0.028	0.031	0.027	0.031	0.033	0.024	0.025
		0.009	0.009	0.011	0.009	0.012	0.016	0.005	0.006
			0.011	0.013	0.011	0.014	0.018	0.007	0.008
				0.012	0.011	0.012	0.016	0.007	0.007
					0.013	0.015	0.02	0.008	0.01
						0.014	0.016	0.008	0.011
							0.019	0.009	0.011
								0.013	0.013

Supplemental Figure S14. Population-pairwise Hudson Fst-estimates. Mean relative genetic distances between pairs of populations.

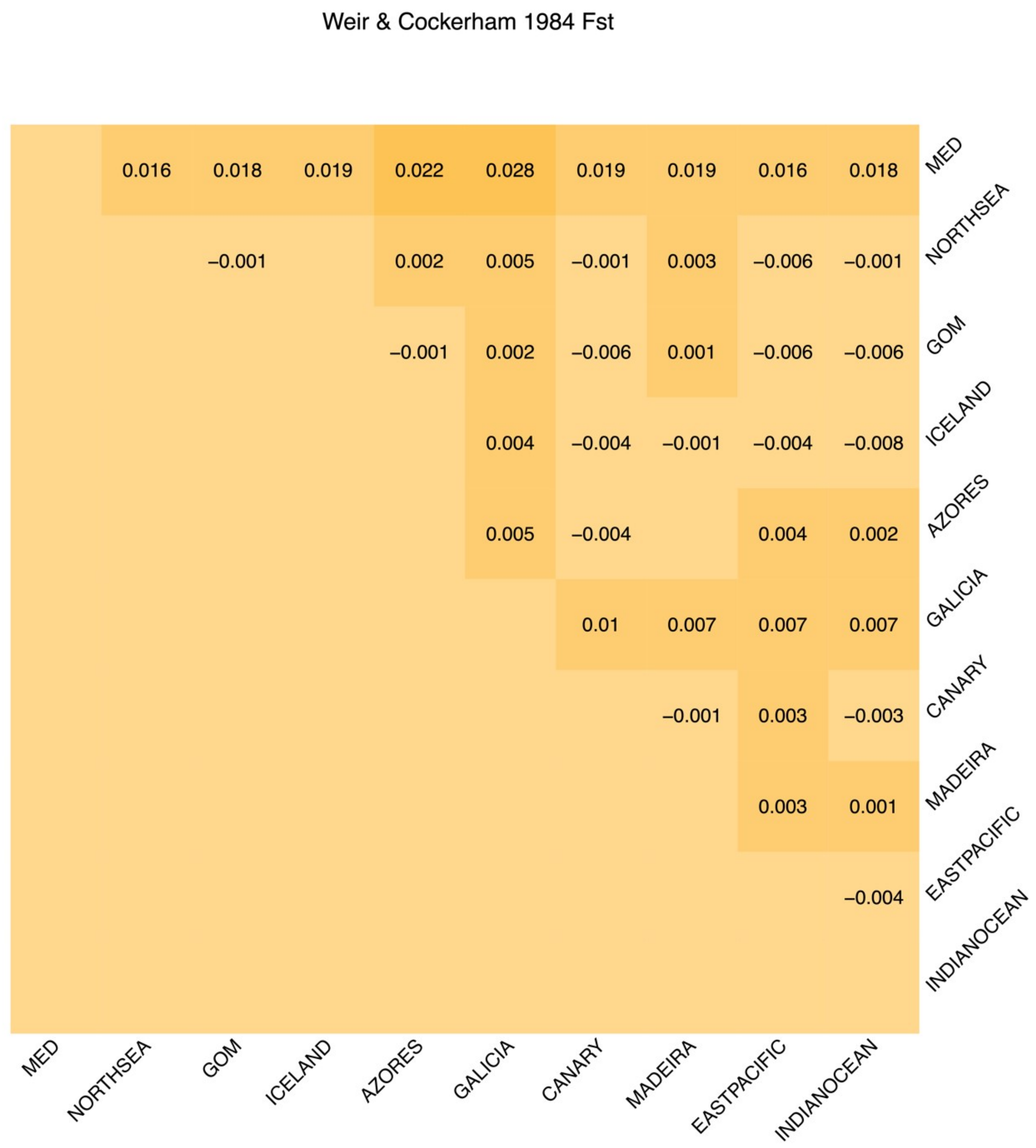

Supplemental Figure S15. Population-pairwise Weir and Cockerhams Fst-estimates. Warmer colors and higher Fst values indicate greater genetic differentiation between populations. The Mediterranean population shows notably higher differentiation compared to other North Atlantic populations.

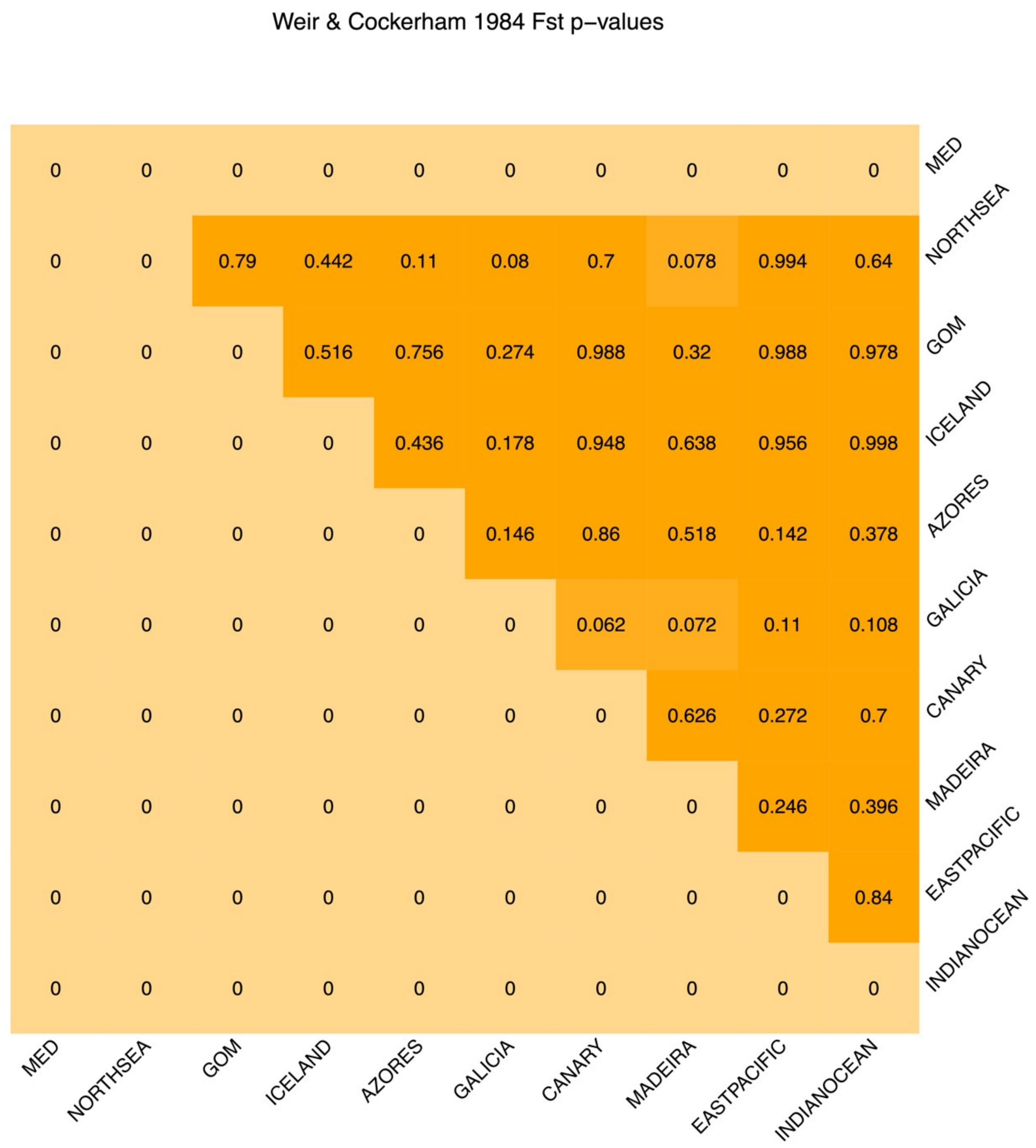

Supplemental Figure S16. Pairwise Fst p-values calculated using the STAMPP package in R with 500 bootstrap replicates. Statistically significant p-values ($p < 0.05$) indicate genetic differentiation between population pairs. The Mediterranean population is significantly different from North Atlantic populations, as reflected by the p-values (0) along the corresponding matrix rows and columns

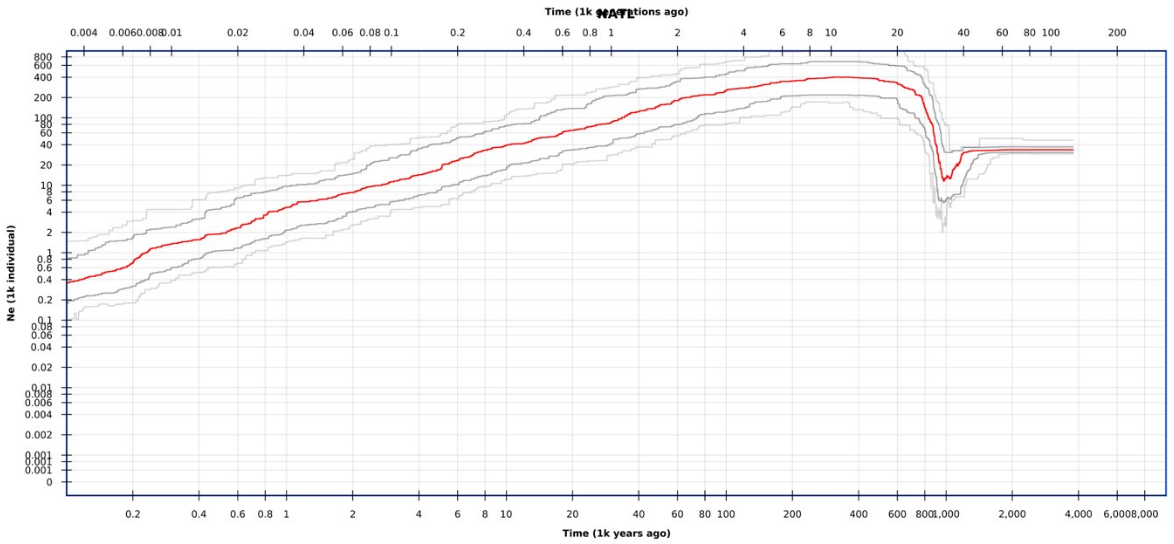

Supplementary Figure S17: Demographic analyses using stairway plots calculated using a mutation rate 2.5x10^{-18} and generation time of 30 years. Red line indicates the median values. Black lines indicate 12.5% and 87.5% percentile values, and grey margins indicate2.5% and 97.5% percentile values.

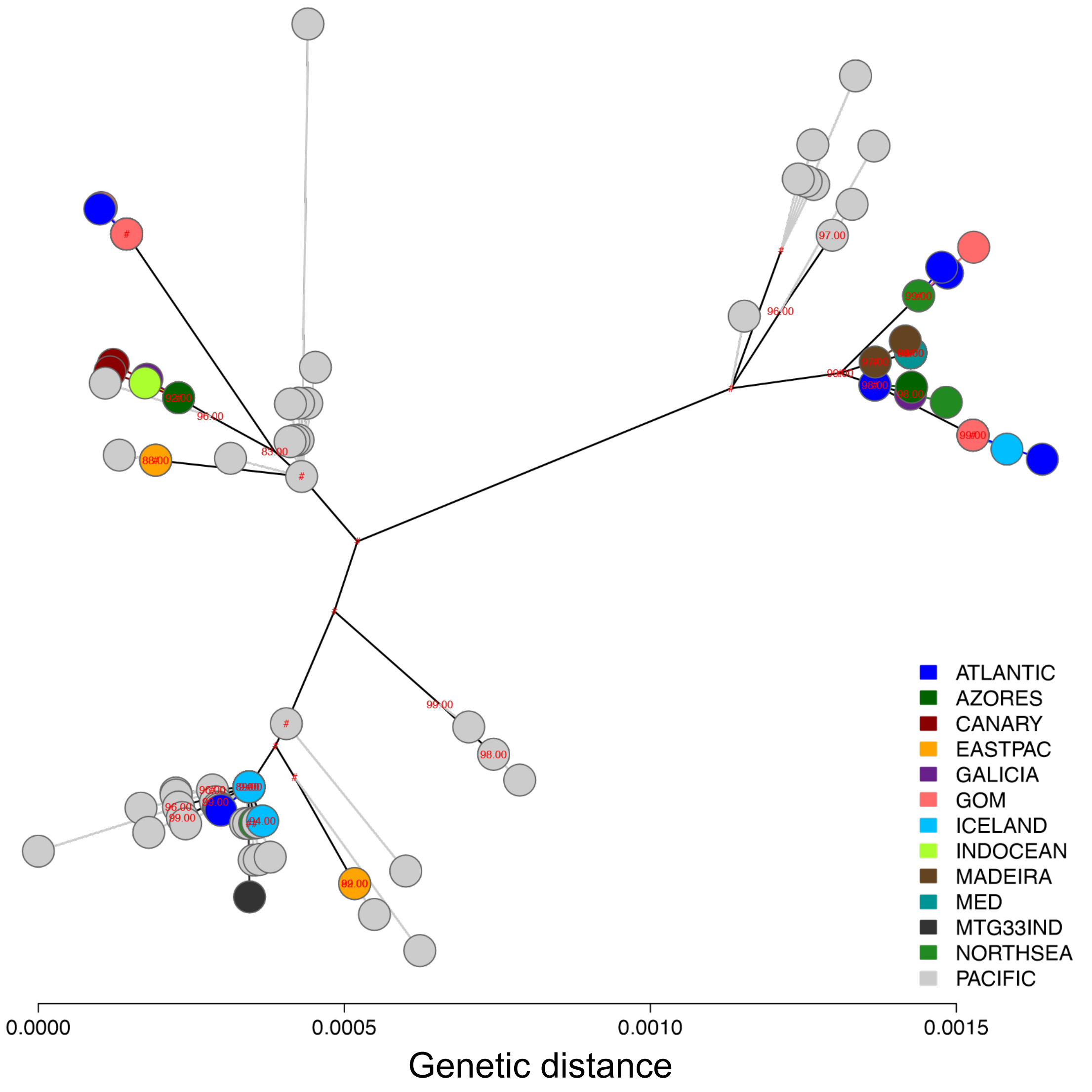

Supplementary Figure S18: Unrooted mitochondrial phylogeny of sperm whales based on the integrated mtDNA dataset using IQ-TREE. Unrooted phylogenetic tree reconstructed from mitochondrial genome sequences, including all of the published mitogenomic haplotypes and the mitochondrial genomes extracted from the present study. Tips are color-coded by sampling region. The tree shows mixed clustering of haplotypes across ocean basins, with no clean ocean-specific clades. This pattern indicates limited matrilineal structure but does not support strong ocean-basin subdivision across the global dataset. Bootstrap support values are shown at major nodes; branch lengths are proportional to genetic distance.

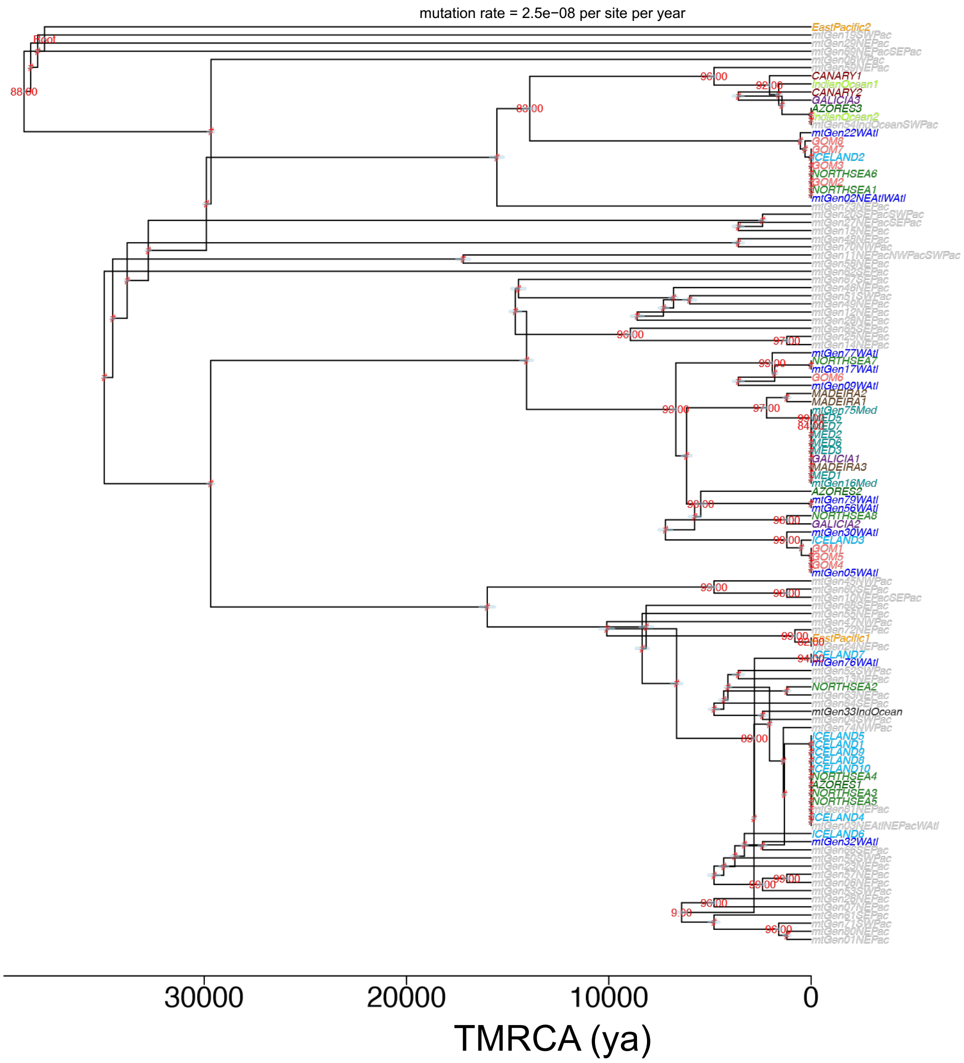

Supplementary Figure S19: Rooted mitochondrial Maximum likelihood phylogeny based on the integrated mtDNA dataset. Rooted mitochondrial phylogeny reconstructed from the same integrated mtDNA dataset and linearized to estimate relative divergence times among haplotypes. Tips are color-coded by sampling region, and node labels indicate bootstrap support.
